# Recent Advances in Atomic-Resolution NMR Investigations of Monoclonal Antibodies

**DOI:** 10.3390/biom16060840

**Published:** 2026-06-08

**Authors:** Béatrice Vibert, Faustine Henot, Oriane Frances, Jérôme Boisbouvier

**Affiliations:** 1 Institut de Biologie Structurale (IBS), Université Grenoble Alpes, CNRS, CEA, 38044 Grenoble, France; 2 Integrated Drug Discovery Department, Sanofi R&D, 94400 Vitry-sur-Seine, France

**Keywords:** NMR, mAbs, Fc, Fab, isotopic labeling, fingerprints, HOS, resonance assignment, methyl groups

## Abstract

Monoclonal antibodies (mAbs) have been the subject of extensive study in recent years due to their recognition as highly promising therapeutic molecules offering high specificity and a low risk of side effects. Monitoring the structure of these molecules is crucial for developing new therapeutics, characterizing interactions with antigens or receptors, and explaining potential changes in activity between antibody production batches. However, commonly used biophysical approaches provide only low-spatial-resolution information, and conventional structural biology techniques such as crystallography and cryo-electron microscopy (cryo-EM) are difficult to apply to these highly dynamic proteins. Solution nuclear magnetic resonance (NMR) spectroscopy is the method of choice for structural studies of flexible proteins at atomic resolution; however, it has traditionally been limited to low-molecular-weight biological systems. In this review, we present recent advances in NMR spectroscopy and advanced isotopic labeling methods that have enabled the atomic-resolution study of both the crystallizable (Fc) and antigen-binding (Fab) fragments of antibodies. We show how NMR is becoming a powerful tool for investigating full-length mAbs at an atomic level, opening up new possibilities for the characterization and in-depth quality control of therapeutic antibodies in solution.

## 1. Introduction

Therapeutic molecules must undergo precise characterization of a wide range of quality attributes throughout their development. Quality control is embedded at every stage of a therapeutic’s life cycle, from initial process development to formulation optimization, stress testing, and long-term storage. With the growing proportion of biomacromolecules compared to small molecules in drug development pipelines, new quality attributes have gained importance. Among these, the higher-order structure (HOS), encompassing the secondary, tertiary and quaternary structures of a biomolecule, is a critical parameter [1]. Within the landscape of protein therapeutics, monoclonal antibodies (mAbs) represent one of the leading classes. Developing innovative tools to assess their HOS and thereby anticipate potential impacts on biological activity or safety, is therefore essential. Indeed, routine analytical techniques currently used for mAb quality control often lack sufficient resolution and are unable to detect subtle yet meaningful changes in HOS. Recently, solution-state nuclear magnetic resonance (NMR) has emerged as a promising approach to characterize antibody structure at atomic resolution under conditions that closely mimic final formulation environments. The development of innovative NMR methods to assess the structure of therapeutic mAbs throughout their life cycle has been strongly encouraged by the U.S. Food and Drug Administration (FDA) [2].

This review presents the latest advances in NMR-based characterization of therapeutic antibodies. Following an introduction to this rapidly expanding class of biologics, we describe the development of simple 2D NMR methods used as fingerprints. While these fingerprints are powerful tools for detecting changes in mAb HOS, they do not provide atomic-resolution information unless individual resonances are assigned to their corresponding amino acids. Achieving such assignments requires isotopically enriched samples. Significant efforts have therefore focused on producing optimally isotope-labeled proteins for NMR studies, including full-length mAbs and their fragments. Recent progress in these production strategies using mammalian cells, yeasts, *E. coli*, and cell-free expression systems is presented. Finally, we highlight the recent backbone and side-chain methyl NMR resonance assignments obtained for antibody fragments and a full-length mAb, which now enable straightforward fingerprints to serve as atomic-resolution tools for HOS characterization. This approach enables detection and precise localization of any structural modification that could occur on a therapeutic antibody. These breakthroughs establish solution-state NMR as a highly informative technique for monitoring the structural integrity of therapeutic antibodies throughout drug development.

## 2. Therapeutic Antibodies: A Fast-Growing Class of Drugs Whose Characterization Is Essential

### 2.1. A Rapidly Expanding Therapeutic Class

Therapeutic antibodies represent a fast-growing and promising class of modern medicines, offering substantial health benefits across a wide range of life-threatening diseases. Cancers, autoimmune disorders, infectious diseases, and chronic inflammations are among the primary indications targeted by therapeutic antibodies [3]. Beyond their expanding therapeutic scope, antibody-based drugs offer major advantages such as high target specificity and generally reduced off-target toxicity compared with traditional small-molecule drugs [4].

**Figure 1:**
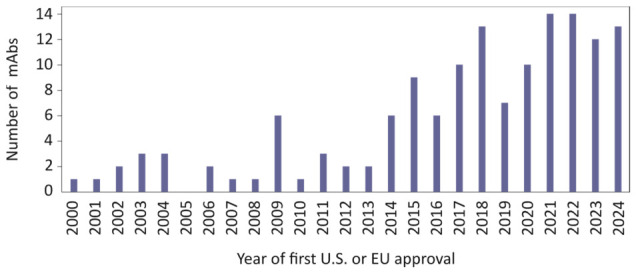
Evolution of the annual number of therapeutic mAbs receiving first approval in the U.S. or EU from 2000 to 2024 [8].

The approval of muromonab-CD3 in 1986 marked a milestone, as it became the first monoclonal antibody (mAb) authorized by the U.S. Food and Drug Administration for the prevention of kidney transplant rejection [5]. Since then, the number of monoclonal antibodies receiving first approval in the U.S. or the EU has risen drastically (Figure 1). Today, approximately 20 new antibodies achieve first approval each year, and more than 200 therapeutic antibodies are currently on the market worldwide [6,7].

### 2.2. Structural Features of IgG1s, the Dominant Therapeutic Subclass

Among the five major human immunoglobulin (Ig) isotypes—IgA, IgD, IgE, IgG, and IgM—IgG is by far the predominant format used in approved and under-development therapeutic antibodies. This preference largely stems from its favorable biochemical and pharmacological properties, including high serum abundance, an extended half-life time, and a broad range of effector functions such as antibody-dependent cellular cytotoxicity and complement activation [3,9]. Within the portfolio of therapeutic antibodies marketed in the United States and Europe, approximately 90% are based on the IgG scaffold. The remaining 10% comprise alternative formats such as antigen-binding fragments (Fabs), single-chain Fragment variable (scFv), and nanobodies [10]. More sophisticated antibody-based drugs derived from the IgG scaffold also exist, such as antibody–drug conjugates and multi-specific antibodies [11].

**Figure 2:**
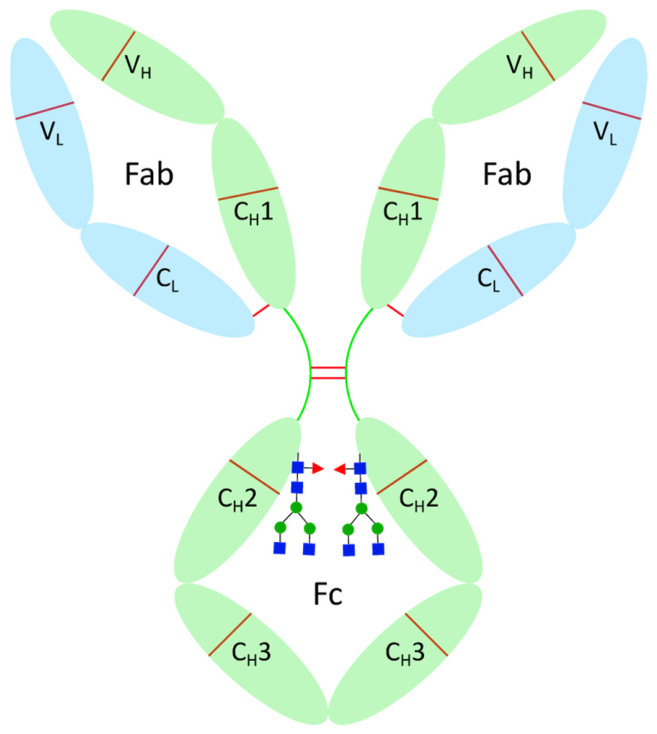
Structural representation of an IgG1 antibody. Disulfide bridges are shown in red, light chains in blue, and heavy chains in green. Glycosylation at N297 of the C_H_2 domains is represented with blue squares for N-acetylglucosamines, green circles for mannoses and red triangles for fucoses.

IgG antibodies are classically depicted with the well-known Y-shaped structure, consisting of two antigen-binding fragments (Fabs) and one crystallizable fragment (Fc). IgGs are roughly 150 kDa proteins composed of four polypeptide chains: two identical heavy chains and two identical light chains. Each heavy chain contains one variable domain (V_H_) and three constant domains (C_H_1, C_H_2, and C_H_3), while each light chain consists of one variable domain (V_L_) and one constant domain (C_L_). The Fab fragment is a heterodimer of approximately 50 kDa formed by the entire light chain and the first two domains (V_H_ and C_H_1) of the heavy chain, held together by both noncovalent interactions and a disulfide bridge. This region is responsible for antigen recognition and provides the specificity of monoclonal antibodies. In contrast, the Fc fragment is a homodimer of approximately 50 kDa composed of the last two domains (C_H_2 and C_H_3) of the heavy chains and is responsible for the interactions with immune-system cells. The two heavy chains are covalently linked through inter-chain disulfide bonds localized at the hinge between the Fab and Fc fragments, together with noncovalent interactions between the two C_H_3 domains (Figure 2).

**Figure 3:**
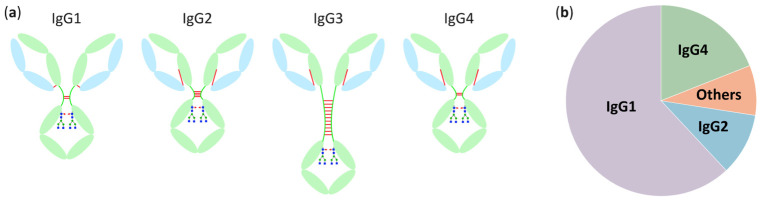
IgG subclasses. (**a**) Schematic representation of IgG subclasses structures. Inter-chain disulfide bridges are shown in red, light chains in blue, and heavy chains in green. (**b**) Distribution of therapeutic mAbs first approved in the U.S. or EU between 2000 and 2024 by IgG subclass [8].

The immunoglobulin G class comprises four subclasses—IgG1, IgG2, IgG3, and IgG4—that share the same overall structure but differ by amino acid sequence within the constant regions of their heavy chains (Figure 3a). The most striking differences arise in the hinge region linking the Fab and the Fc fragments: it spans 12 amino acids in IgG2 and IgG4, 15 in IgG1, and 62 in IgG3. Additionally, the number of disulfide bridges between the two heavy chains varies substantially across subclasses—2 in IgG1 and IgG4, 4 in IgG2, and 11 in IgG3. Another structural difference between IgG subclasses lies in the position of the disulfide bond connecting the light and heavy chains within the Fab fragment. This inter-chain disulfide bond links the C-terminal cysteine of the light chain, located in the C_L_ domain, to a cysteine in the C_H_1 domain of the heavy chain. In IgG1, this cysteine is found at position 220, whereas in IgG2, IgG3, and IgG4 it is located at position 131. Despite these positional differences, the two residues are spatially close, and the nature of this linkage does not appear to influence the overall structure or function of the antibody [12]. The variations in hinge length, disulfide connectivity, and overall sequence confer distinct degrees of flexibility and proteolytic resistance to each subclass [9]. These structural distinctions translate directly into functional differences, particularly in their ability to interact with Fc gamma receptors (FcγRs) and activate the complement system via C1q binding. Consequently, only IgG1, IgG2, and IgG4 subclasses have been broadly adopted for human therapeutic use, due to their extended serum half-life time, consistent biophysical behavior, and well-characterized functional profiles. Among them, IgG1 is by far the most commonly used subclass in monoclonal therapeutic antibodies, owing to its strong effector functions and broad clinical versatility (Figure 3b) [10]. It has also been shown that IgG1, IgG4, and IgG2 with identical variable regions have decreasing Fab-Fab and Fab-Fc flexibility, and that IgG1 is the most stable isotype against aggregation, possibly because of increased repulsive protein–protein interactions [13,14].

IgG antibodies possess multiple functional properties that enable a wide range of therapeutic mechanisms. Through the antigen-binding Fab region, IgGs can inhibit ligand–receptor interactions, neutralize antigens, or activate specific pathways depending on the target. In parallel, the Fc domain mediates several immune effector functions by engaging Fc gamma receptors or complement proteins. These Fc-dependent mechanisms include antibody-dependent cellular cytotoxicity (ADCC), antibody-dependent cellular phagocytosis (ADCP), and complement-dependent cytotoxicity (CDC), and contribute to the elimination of target cells and modulation of immune responses [15]. Both ADCC and ADCP rely on antibodies binding to antigens on the surface of target cells, after which the Fc region of these antibodies is recognized by immune cells through Fcγ receptors. In ADCC, the main effector cells are natural killer cells, which, once activated, release cytotoxic molecules that trigger apoptosis of the target cells. In contrast, ADCP is driven by phagocytic cells such as macrophages, which engulf and digest the antibody-coated cells. CDC involves activation of the complement cascade through the binding of C1q to the Fc region of antibodies attached to antigens. This interaction triggers a series of complement reactions that ultimately form the membrane attack complex, which creates pores in the target cell membrane, leading to cell lysis. These mechanisms are fundamental to the biological activity of therapeutic antibodies and can be modulated during antibody development through engineering of the antibody sequence.

Therapeutic antibodies depend on proper folding and post-translational modifications to ensure activity and low immunogenicity, making mammalian cells the system of choice for recombinant antibody production [16]. Approximately 80% of therapeutic antibodies are manufactured using Chinese Hamster Ovary (CHO) cells, which provide human-like glycosylation, thereby reducing the risk of adverse immune responses [17].

### 2.3. IgG Glycosylation: A Key Post-Translational Modification Impacting Biological Activity

**Figure 4:**
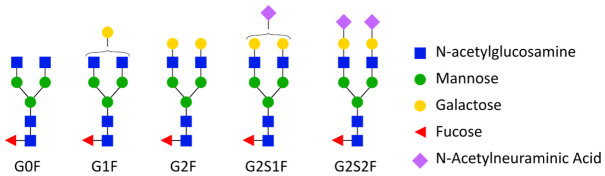
Typical glycan structures found in therapeutic mAbs at N297 glycosylation sites.

IgG antibodies contain a conserved N-glycosylation site at asparagine 297 (N297) on each heavy chain, located within the C_H_2 domains of the Fc fragment. Fc N-glycosylation is introduced through a series of enzymatic steps occurring in the endoplasmic reticulum (ER) and the Golgi apparatus. In the ER, the entire preassembled oligosaccharide is transferred onto N297 of the nascent heavy chain. Following initial trimming of glucose and mannose residues, the light and heavy chains assemble to form the intact immunoglobulin. Once correctly folded, the antibody undergoes further glycan maturation in the Golgi, where various glycosyltransferases and glycosidases introduce or remove sugar units, generating a mixture of diverse glycoforms (Figure 4) [15,18].

Numerous studies have demonstrated that both the presence and the specific composition of Fc glycans profoundly influence antibody structure, stability, effector functions, and even immunogenicity. Glycosylation modulates the interaction of the Fc region with Fcγ receptors and the complement component C1q, thereby shaping key mechanisms such as ADCC and CDC [19]. One of the most critical glycan features is core fucosylation. ADCC activity is highly sensitive to the level of fucose attached to the Fc oligosaccharide: lower fucosylation enhances binding to FcγRIIIa and increases cytotoxic potency [20]. In IgG1 antibodies, afucosylation has been shown to improve FcγRIIIa affinity by up to 50-fold, resulting in markedly enhanced ADCC activity [21]. Another glycan residue with strong functional impact is terminal galactose. Increased galactosylation correlates with elevated CDC activity, largely due to enhanced binding of the C1q protein to the Fc domain. Higher galactose content therefore promotes more efficient complement activation and target-cell lysis [22]. These two major effects demonstrate the influence of the glycosylation pattern on the biological activity of therapeutic antibodies and highlight the need for precise and systematic control of glycosylation profiles. Indeed, the size and composition of the glycans directly impact the structure of the crystallizable fragment around N297, which lies in close proximity to the receptor-binding sites involved in immune reactions.

### 2.4. Higher-Order Structure as a Critical Quality Attribute

mAbs are large, structurally complex biomolecules, and their rapid expansion in therapeutic applications presents significant challenges for comprehensive characterization throughout development and manufacturing. Ensuring their efficacy and safety requires tight control of multiple critical quality attributes (CQAs), including glycosylation patterns, aggregation levels, chemical modifications such as oxidation, and higher-order structure. HOS refers to the three-dimensional organization of the antibody, including its secondary, tertiary, and quaternary structures, which collectively determine the molecule’s conformation, stability, and biological activity. Even subtle alterations in HOS can impair essential functions such as antigen binding or Fc-mediated interactions with effector receptors. Improper folding can result in off-target binding or no binding to the target. Structural disruptions may also lead to aggregation, representing a loss of proper folding, which can severely impact both therapeutic efficacy and patient safety. HOS also contributes to formulation stability and viscosity, which are essential parameters that must be controlled during therapeutic mAb development. Higher-order structure is also a critical parameter when comparing molecules to determine whether two therapeutics are biosimilars. Moreover, many chemical modifications known to affect antibody activity (e.g., deamidation or oxidation) often induce changes in tertiary or quaternary structure. Consequently, rigorous assessment of mAb HOS is critical across all stages of development and production to ensure drug performance and patient safety.

**Figure 5:**
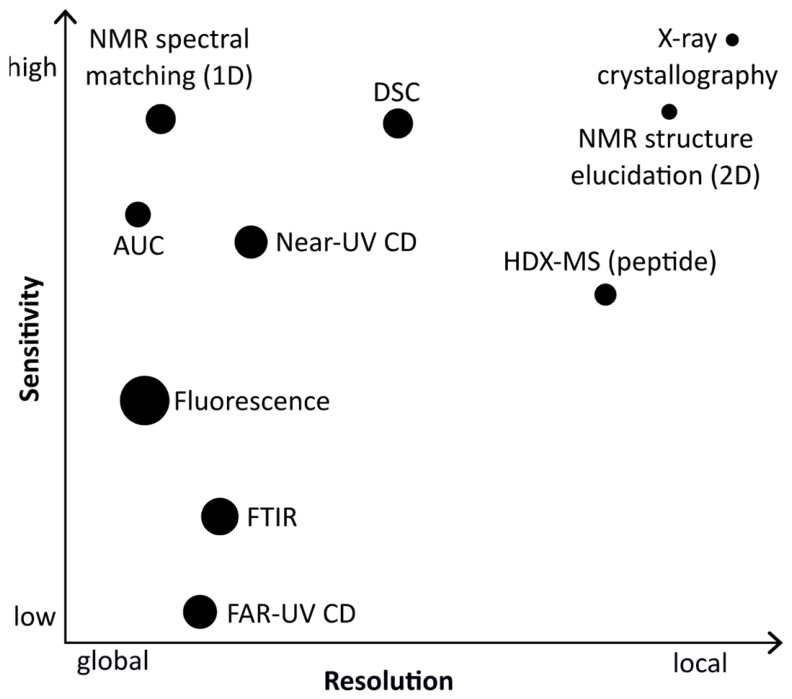
Schematic representation of the resolution and sensitivity of techniques used for HOS characterization. Each technique is depicted as a sphere, where the sphere size reflects the associated resource requirement (larger spheres indicate lower resource demands). Adapted from https://www.sciencedirect.com/science/article/pii/S0022354916416953?via%3Dihub (accessed on 22 January 2026) [1].

Various biophysical techniques, such as far- and near-ultraviolet circular dichroism (CD), dynamic light scattering (DLS), Fourier-transform infrared spectroscopy (FTIR), intrinsic fluorescence, and differential scanning calorimetry (DSC), are routinely employed to assess the HOS of therapeutic monoclonal antibodies. However, these methods provide only low-spatial-resolution information (Figure 5). They detect global conformational changes but cannot identify the specific domains or residues responsible for the observed signal variations. Moreover, these techniques do not allow the detection of subtle structural modifications that may nevertheless impact biological activity. Hydrogen-deuterium exchange mass spectrometry (HDX-MS) offers higher structural resolution, as it detects local conformational changes by probing solvent accessibility at the peptide or even the residue level. Nevertheless, HDX-MS has notable limitations in the context of therapeutic mAb quality control. The sample preparation process, including deuterium exchange and proteolytic digestion, must be performed under non-native, low-pH and low-temperature conditions. As a result, HDX-MS cannot fully characterize antibody structure as it exists in formulation buffer and provides limited insights into precise tertiary or quaternary structural arrangements [23]. High-resolution structural techniques such as X-ray crystallography and cryo-electron microscopy (cryo-EM) are widely used for protein structure determination. However, these methods are not adapted to assess antibody HOS in solution. First, the need to crystallize or freeze the protein can alter or constrain its tertiary structure, providing static snapshots that do not fully capture the conformational behavior of monoclonal antibodies under formulation conditions. Moreover, the inherent flexibility and dynamic nature of mAbs are a major challenge for structural characterization by cryo-EM, often limiting achievable resolution. Finally, the sample preparation workflows required for both crystallography and cryo-EM are time-consuming, technically demanding, and therefore not compatible with routine characterization of therapeutic mAbs [24].

Liquid-state NMR offers the possibility of characterizing the HOS of monoclonal antibodies at atomic resolution, under formulation conditions, with minimal sample preparation. Historically, however, protein NMR has been constrained by the large molecular size of antibodies, making high-resolution structural analysis extremely challenging. Recent advances in NMR spectroscopy, protein production, and isotopic labeling have significantly expanded the applicability of NMR to complex biomolecules, giving it the potential to become a method of choice for HOS characterization of therapeutic mAbs throughout their development [25]. A recent collaborative study between the FDA and Bruker further demonstrated “the power and sensitivity of NMR in assessing the structural integrity of formulated mAbs and their biosimilars, especially under stress conditions” [2] (p. 17), emphasizing the relevance of NMR for regulatory agencies at all stages of drug development. This work not only highlights the suitability of NMR for detecting subtle conformational differences but also underscores the substantial technical requirements associated with the method, such as advanced instrumentation, and expert-level data interpretation. These considerations explain the growing number of research efforts focused on expanding the use of NMR for mAb HOS analysis.

In order to be suitable for assessing the HOS of monoclonal antibodies throughout therapeutic development, a method must require minimal sample preparation, provide results rapidly, and allow straightforward interpretation. In the context of antibody NMR, this translates into using samples at natural isotopic abundance, recording spectra directly in solution under conditions as close as possible to their formulation buffer, with experiment times that ideally do not exceed a single day. Simple 1D ^1^H or heteronuclear 2D ^1^H-^13^C and ^1^H-^15^N experiments can be acquired with acceptable sensitivity on therapeutic mAbs due to the high protein concentrations typically present in their formulations. The resulting spectra can then be compared to structural fingerprints, as each signal reflects the local chemical environment of the observed nuclei and, collectively, the atomic-level structure of the antibody. Even without identifying the amino acid corresponding to each signal, NMR fingerprints can still offer highly sensitive insights into the HOS of mAbs. Consequently, such fingerprint-based NMR approaches have been extensively explored and developed as promising tools for routine HOS characterization of therapeutic antibodies.

## 3. NMR Fingerprinting as a Powerful Tool for HOS Characterization of mAbs at Natural Abundance

Protein NMR typically focuses on the magnetically active nuclei ^1^H, ^13^C, and ^15^N, which occur at natural isotopic abundances of 99.99%, 1.11%, and 0.36%, respectively. The main experiments used to obtain NMR fingerprints of monoclonal antibodies are 1D ^1^H, 2D ^1^H-^15^N, and 2D ^1^H-^13^C methyl spectra. In classical 1D ^1^H experiments, essentially every proton in the protein contributes to a signal. In contrast, 2D ^1^H-^15^N spectra primarily detect backbone amide NH groups, whereas methyl 2D ^1^H-^13^C spectra target methyl groups present in the side chains. For mAbs at natural isotopic abundance, these experiments yield approximately 5000 signals in ^1^H spectra, around 600 in ^1^H-^15^N spectra, and about 300 in ^1^H-^13^C methyl spectra. The resulting high density of resonances leads to substantial spectral overlaps, complicating data interpretation. The large molecular size of antibodies (approximately 150 kDa) also induces slow molecular tumbling in solution, resulting in fast relaxation rates and producing broader, less resolved signals. To address these limitations, a widely adopted solution is the “divide-and-conquer” strategy, in which the Fab and Fc fragments (approximately 50 kDa each) are analyzed separately. In IgG1 antibodies, these fragments are connected by a flexible hinge region, which allows them to behave as structurally independent domains and therefore enables their study separately. Considering these smaller proteins significantly improves NMR spectral resolution and facilitates more detailed structural characterization.

### 3.1. A “Divide-and-Conquer” Strategy

**Figure 6:**
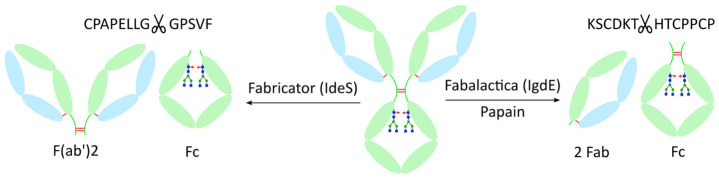
Antibody fragments resulting from IgG digestion below (left) or above (right) disulfide bridges. Inter-chain disulfide bridges are shown in red, light chains in blue, and heavy chains in green.

Two main strategies are commonly used to obtain Fab and Fc fragments. The first involves cleaving the antibody below the disulfide bridges of the hinge using the IdeS enzyme (FabRICATOR^®^), an IgG-specific cysteine protease that generates F(ab’)_2_ and Fc fragments. The second strategy targets the cleavage above the disulfide bridges, typically using papain or a cysteine protease such as an IgdE enzyme (FabALACTICA^®^), to obtain Fab and Fc fragments (Figure 6). These two approaches have been widely employed to analyze isolated Fab and Fc fragments by NMR, leading to a substantial improvement in spectral quality [26,27,28].

### 3.2. 1D ^1^H NMR for Rapid Characterization of mAbs

Due to the high natural abundance and gyromagnetic ratio of ^1^H, one-dimensional ^1^H NMR is the most sensitive NMR technique for characterizing protein HOS. A rapid 1D ^1^H experiment readily reports on the folding state of a monoclonal antibody through the dispersion of the amide proton signals, and on its aggregation state through the overall signal intensity. This approach is therefore well suited for formulation development, stability studies, and forced-degradation assays on mAbs. 1D ^1^H spectra can be compared either manually, by identifying chemical shift perturbations or signal intensity losses, or by using statistical tools that allow the comparison of large datasets and the detection of subtle variations in the 1D fingerprint.

Aggregation can be easily monitored by 1D ^1^H NMR, as it causes enhanced relaxation due to increased particle size, resulting in broader peaks and reduced signal intensity. Since the methyl region is known to reflect the apparent structural integrity of mAbs, the spectral window from approximately −0.5 to 2 ppm is commonly examined. For example, Bramham and coworkers investigated mAb aggregation during liquid–liquid phase separation (LLPS) and demonstrated by ^1^H NMR that aggregation observed within the dense phase is reversible upon dilution, indicating that LLPS could serve as a strategy to concentrate mAb solutions in the pharmaceutical industry [29,30]. The 1D ^1^H NMR method has therefore proven particularly useful for optimizing protein formulations, as signal intensity directly reflects the stability and oligomeric state of the mAb solution [31,32].

Numerous studies have focused on using 1D ^1^H fingerprints to detect changes in antibody solutions during forced-degradation tests, including UV-stress experiments [33] and pH-dependent studies [32,34]. 1D ^1^H NMR has also shown strong performance in comparing HOS similarity across different lots of formulated drug products, revealing differences in oligomerization and folding states [35].

Despite these successful applications, 1D ^1^H NMR of mAbs is not always straightforward, as most formulations contain additives whose intense NMR signals overlap with those of the mAb. To overcome this limitation and enhance the structural information obtained from 1D ^1^H spectra, several groups have proposed exploiting spin diffusion among protons in well-structured regions. This selectively emphasizes HOS-related spectral components while reducing contributions from excipients and mobile regions. The method, referred to as PROFILE (protein fingerprint by line-shape enhancement), yields a fingerprint focused on the structural characteristics of mAbs [25,34,36,37].

Despite the simplicity and inherent sensitivity of 1D ^1^H NMR fingerprints, their potential for mAb HOS characterization remains limited because of the poor structural resolution they provide. These spectra mainly report on the global structure of the antibody, and no residue- or atom-specific information can be extracted due to extensive signal overlaps. In contrast, multidimensional heteronuclear NMR experiments offer more detailed fingerprints in which most resonances are individually resolved. Among these, 2D ^1^H-^13^C and ^1^H-^15^N correlation spectra have been extensively developed and applied to achieve a more precise characterization of mAb HOS.

### 3.3. 2D ^1^H-^15^N Spectra, the Gold Standard for Protein Characterization by NMR

Two-dimensional ^1^H-^15^N correlation spectra are often considered as the gold standard for protein characterization by NMR. Because these spectra typically display one signal per residue (except for prolines), ^1^H-^15^N NMR fingerprints provide residue-level information on the chemical environment of the protein backbone and are therefore highly sensitive reporters of the protein structure. Such spectra are widely used in biochemical NMR to detect chemical modifications, structural rearrangements, ligand binding, or interactions with molecular partners. Even without backbone resonance assignments, 2D ^1^H-^15^N fingerprints can reveal structural differences between samples.

A major limitation of using 2D ^1^H-^15^N spectra rather than 1D ^1^H spectra to study proteins at natural abundance is the inherently low sensitivity of these experiments. The very low natural abundance of ^15^N (0.36%) drastically affects the sensitivity of 2D ^1^H-^15^N measurements. Sensitivity is further compromised when working with large molecules such as 150 kDa antibodies, due to additional relaxation occurring during magnetization transfer steps. Despite these challenges, several research groups have successfully explored the use of natural-abundance ^1^H-^15^N fingerprints to investigate mAb HOS [27,38,39,40]. Multiple approaches have been explored to improve the quality of 2D ^1^H-^15^N spectra of antibodies at natural abundance. On the experimental side, TROSY (Transverse Relaxation-Optimized SpectroscopY) pulse sequences were designed to enhance spectral quality for large, isotopically labeled proteins, but they provide only modest advantages for fully protonated 150 kDa mAbs. Because antibodies are highly stable proteins with elevated unfolding temperatures, NMR spectra can be acquired at temperatures up to 50 °C. Such high temperatures reduce transverse relaxation, thereby improving both resolution and sensitivity compared to the typical 5–35 °C range used in protein NMR. To obtain high-quality 2D ^1^H-^15^N spectra of mAbs at natural abundance within reasonable acquisition times, the SOFAST-HMQC (band-Selective Optimized-Flip-Angle Short Transient Heteronuclear Multiple Quantum Coherence) experiment has emerged as the most effective option [27,39]. This approach relies on the selective excitation of amide protons, leaving a large reservoir of unperturbed aliphatic protons that facilitates rapid return of the amide ^1^H-^15^N spin systems to equilibrium through efficient T_1_ relaxation. The accelerated recovery enables shorter recycle delays, significantly reducing acquisition time and allowing more scans to be collected within the same total measurement time, thus improving sensitivity [41].

Only a few 2D ^1^H-^15^N spectra recorded on full monoclonal antibodies at natural abundance have been reported, but these studies demonstrate that such fingerprints can indeed be acquired. Notably, a ^1^H-^15^N SOFAST-HMQC spectrum acquired for 68 h using 20 mg of antibody as the starting material showed reasonable sensitivity and displayed many well-resolved signals in the peripheral regions of the spectrum out of the 650 expected signals [27]. However, even at 50 °C and using a 900 MHz spectrometer, the central region of the spectrum remains heavily overlapped, highlighting the limitations of 2D ^1^H-^15^N NMR for HOS characterization of intact mAbs at natural abundance. Reducing the size of the protein through enzymatic digestion of the mAb offers a straightforward strategy to enhance the applicability of ^1^H-^15^N fingerprints for HOS studies. Arbogast and co-workers compared the ^1^H-^15^N SOFAST-HMQC spectra of an intact mAb, a non-purified mixture of Fab and Fc fragments, and the two purified fragments. The strong correspondence between the intact mAb spectrum and that of the fragment mixture validated the “divide-and-conquer” approach. Furthermore, because the spectra of the isolated Fab and Fc contain approximately 65% and 35% of the total signals expected for the full mAb, respectively, both fragments exhibit substantially reduced spectral overlaps. In addition, the resolution of the full mAb spectrum is primarily limited by transverse relaxation, which increases with protein size, and is thereby particularly pronounced in a fully protonated protein of 150 kDa. When analyzing the 50 kDa fragments, the signals relax more slowly, resulting in narrower linewidths. This effect accounts for the significant improvement in resolution and the corresponding increase in signal-to-noise ratio. As a result, the ^1^H-^15^N fingerprints of the individual Fab and Fc domains offer far clearer structural information than those of the intact mAb [27,39].

These fingerprints of mAb fragments can then be used to compare different antibodies or fragments and to detect structural changes that directly reflect the HOS of the full-length mAb. As a proof of concept, Hodgson and co-workers compared the Fc fragments of several commercial IgG1 antibodies using ^1^H-^15^N fingerprinting [40]. However, due to both the low natural abundance and the low gyromagnetic ratio of ^15^N, ^1^H-^15^N SOFAST-HMQC spectra acquired on mAbs or their fragments at natural abundance suffer from limited sensitivity. Obtaining high-quality ^1^H-^15^N fingerprints typically requires several days of acquisition on very high-field spectrometers equipped with cryogenic probes, making this approach poorly suited for routine HOS assessment during therapeutic antibody development.

### 3.4. 2D ^1^H-^13^C Methyl Fingerprints as Sensitive HOS Reporters

**Figure 7:**
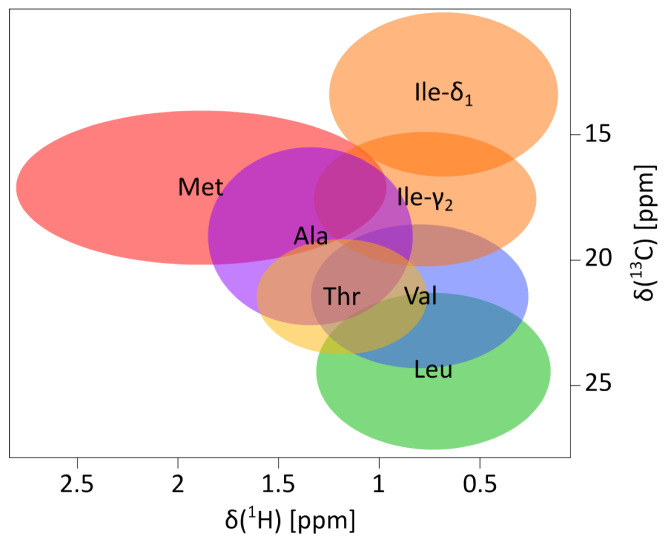
Statistical distribution of ^1^H-^13^C methyl signals per amino acid type. Signals from both methyl groups of leucines (δ_1_ and δ_2_) and valines (γ_1_ and γ_2_) are included in the Leu and Val regions, respectively. The boundaries of the colored ellipses demonstrate the second standard deviations. Adapted from [42].

A common alternative to 2D ^1^H-^15^N amide spectra for obtaining structural information on large proteins at natural abundance is 2D ^1^H-^13^C methyl spectroscopy. Six out of the 20 amino acids, alanine, isoleucine, leucine, methionine, threonine, and valine, contain one or two methyl groups in their side chains, which can be considered as excellent NMR probes. While the presence of three equivalent protons increases signal intensity, the side-chain flexibility and the rapid rotation of methyl groups located at the end of side chains lead to more favorable relaxation properties, resulting in sharper signals. Also, the higher natural abundance of ^13^C relative to ^15^N results in higher sensitivity. Furthermore, the ^1^H and ^13^C resonances of methyl groups fall within a well-defined region of the spectrum, with minimal overlap with other aliphatic protons. This spectral window also differs between amino acids, which further facilitates fingerprint analysis (Figure 7).

**Figure 8:**
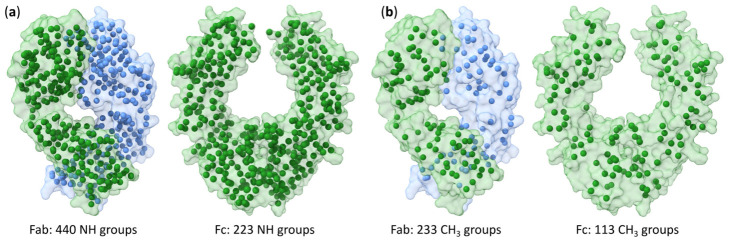
Representation of amide and methyl groups in Fab and Fc fragments of the NISTmAb. (**a**) Amide groups are represented by spheres. (**b**) Methyl groups are represented by spheres. Light chains are shown in blue and heavy chains in green. Crystal structures of NISTmAb Fc (PDB 5VGP) and Fab (PDB 5K8A) fragments are shown.

Because methyl groups are sensitive to the hydrophobic packing of proteins, they serve as excellent reporters of protein folding and overall structure. Consequently, 2D ^1^H-^13^C methyl NMR fingerprinting represents a valuable alternative to ^1^H-^15^N fingerprinting for the HOS characterization of mAbs. Methyl groups are less abundant than NHs in antibodies, with approximately 30% of amino acids containing at least one methyl group, resulting in NMR fingerprints with fewer signals and therefore fewer overlaps. Moreover, they are well distributed throughout the structure of mAbs, ensuring that both the Fab and the Fc fragments are well represented by methyl group probes (Figure 8). Therefore, any structural modification occurring in the mAb will alter the chemical environment of at least one methyl-containing amino acid, leading to a corresponding signal shift in the NMR methyl fingerprint.

**Figure 9:**
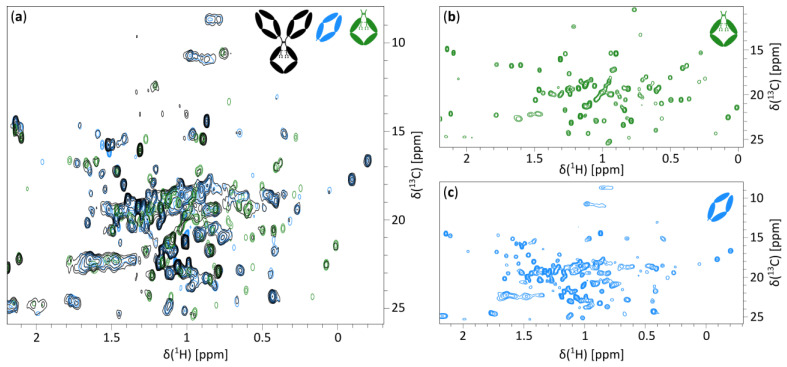
^1^H-^13^C ALSOFAST methyl spectra of isatuximab full-length mAb (black), Fc (green), and Fab (blue). Spectra were recorded for 9 h on an 800 MHz spectrometer at 37 °C. Fc and Fab samples were obtained from isatuximab digestion using the FabRICATOR^®^ enzyme. (**a**) Overlay of methyl spectra of mAb, Fab, and Fc. (**b**) Methyl spectrum of Fc. (**c**) Methyl spectrum of Fab.

Arbogast and co-workers demonstrated for the first time the feasibility of using 2D ^1^H-^13^C methyl NMR fingerprinting for structural mapping of an intact mAb at natural isotopic abundance [26]. A gradient-selective sensitivity-enhanced heteronuclear single quantum coherence (gsHSQC) experiment optimized for the methyl region was recorded for 12 h using a 900 MHz spectrometer on a mAb at 50 °C, resulting in a spectrum “amenable to detailed assignment and analysis” [26] (p. 3558). About 60% of the expected signals gave reasonably resolved peaks, showing both the strength and the limits of methyl NMR fingerprinting in characterizing the structure of intact mAbs at natural abundance. At natural abundance, approximately 350 signals are expected in a 2D methyl spectrum of a full-length antibody. Using enzymatic digestion to study Fab and Fc fragments separately not only reduces signal overlap but also produces sharper signals (Figure 9a). As a result, the Fab and Fc spectra display increased sensitivity and resolution, with most of the signals being well resolved (Figure 9b,c).

This divide-and-conquer approach has been widely used to improve the spectral quality of mAbs [26,27,28,39,40,43,44]. With the same objective of enhancing the quality of methyl fingerprints of mAbs and their fragments while reducing acquisition times to a few hours, several studies have focused on comparing and adapting pulse sequences, as well as optimizing the entire workflow, from sample preparation to data analysis. Most of this work has been performed on the NISTmAb, an IgG1 antibody developed by the National Institute of Standards and Technology as a reference material for evaluating analytical methods used to determine the physicochemical and biophysical attributes of monoclonal antibodies, and extensively used in NMR studies [26,27,36,38,39,45,46,47,48,49,50,51,52,53,54,55].

Using the SOFAST-HMQC pulse sequence provides the same advantages for ^1^H-^13^C methyl spectra as for ^1^H-^15^N amide spectra, resulting in reduced acquisition times. An alternative experiment, called ALSOFAST [56], based on the same principle as SOFAST but using a different excitation scheme for the magnetization of interest, has also been considered for acquiring 2D spectra of antibodies. This NMR experiment was further optimized and adapted for very large, fully protonated proteins, resulting in the XL-ALSOFAST experiment [57]. This pulse sequence relies on (i) a reduced heteronuclear transfer period before the detection associated with delayed decoupling to minimize signal losses arising from transverse relaxation, as well as (ii) improved gradient selection to better suppress background signals. Consequently, the XL-ALSOFAST experiment exhibits increased sensitivity and enhanced artifact suppression. When applied to a fully protonated antibody, the resulting spectrum showed a two- to three-fold increase in sensitivity compared with standard HSQC experiments. In addition, non-uniform sampling (NUS) enables a further decrease in experimental time, with methyl spectra acquired using samples at 20 mg/mL of Fab or Fc at natural isotopic abundance in as little as 30 min using 50% NUS while maintaining sensitivity and peak-position accuracy [26,39]. Such optimization demonstrates that NMR fingerprinting is no longer limited by acquisition time and can be implemented routinely as a quality control tool for therapeutic mAbs.

To further support the implementation of 2D-NMR methods in routine workflows for biotherapeutic mAb development, an interlaboratory study involving 26 laboratories from industry, government, and academia was conducted [38,55]. Both 2D ^1^H-^15^N and ^1^H-^13^C spectra were acquired on the NISTmAb Fab and reproducibility across laboratories and spectrometers was assessed. The study concluded that 2D-NMR approaches, especially ^1^H-^13^C methyl fingerprinting, can be used as routine measurements to assess the HOS of mAbs with high precision. Even in the absence of residue-specific assignments, each signal in the 2D spectrum serves as a reporter of the local structural environment at atomic resolution.

**Figure 10:**
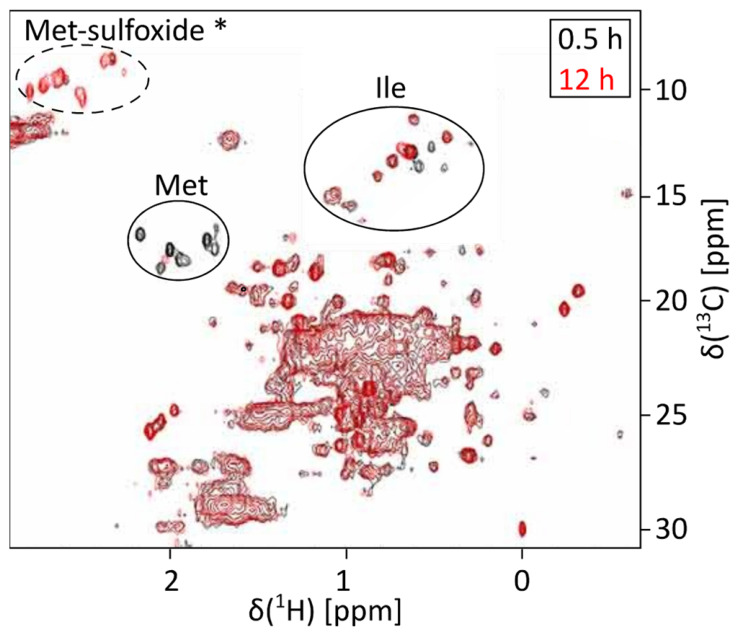
Overlay of 2D ^1^H-^13^C methyl gHSQC spectra of NISTmAb at natural abundance after both 0.5 h (black) and 12 h (red) of oxidation with hydrogen peroxide. Spectra were recorded at 600 MHz and 50 °C. Signals corresponding to methionine sulfoxide residues are highlighted with a dotted black circle. The asterisk indicates that these signals are folded in the indirect dimension and are expected to appear around 34 ppm under these conditions. Adapted from [53].

Several recently reported studies have focused on using ^1^H-^13^C methyl NMR spectra acquired on mAbs or their fragments at natural isotopic abundance to assess the HOS of therapeutic antibodies. These studies aimed at detecting aggregation, structural differences between glycoforms, formulation-induced effects, and structural changes triggered by forced deamidation or oxidation, using methyl fingerprint comparisons. For example, Majumder and co-workers applied methyl NMR fingerprinting to a series of Pfizer mAbs to examine correlations between HOS and storage stability. The fingerprinting approach, applied to individual fragments, enabled a clear distinction between well-behaved mAbs and those prone to aggregation [28]. In another study, they used ^1^H-^13^C HSQC spectra of mAb fragments to evaluate the impact of forced deamidation on the HOS. The Fc methyl fingerprint showed only minimal changes following deamidation, demonstrating that this chemical modification does not perturb the overall Fc structure. Likewise, no significant chemical-shift differences were observed for Fab fragments, in agreement with cell-based assay results (Figure 10) [43].

Chemical oxidation is another commonly applied forced-degradation method. In the context of developing NMR tools to monitor mAb HOS as a quality attribute during drug development, 2D ^1^H-^13^C methyl fingerprints were recorded on the full-length NISTmAb at natural isotopic abundance at different levels of H_2_O_2_-induced oxidation [53]. In addition to chemical-shift changes for methionine residues, additional perturbations were detected by overlaying 2D spectra, including shifts in the isoleucine region. These observations demonstrate that 2D methyl fingerprinting can sensitively detect HOS modifications and highlight the value of residue-specific assignment for achieving atomic-level structural interpretation.

During mAb development, optimization of the formulation is essential. Factors such as mAb concentration and excipients can affect protein structure, potentially reducing biological activity. To assess the impact of two surfactants, polysorbate 20 (PS20) and polysorbate 80 (PS80) in mAb formulations, 2D ^1^H-^13^C methyl NMR spectra were recorded on Fab and Fc fragments of a Pfizer IgG1 [58]. Cross-peak volumes and chemical shifts were compared across increasing concentrations of PS20 and PS80 for both fragments. While no significant changes were observed for the Fc, the Fab spectra displayed a marked decrease in cross-peak volumes with increasing concentrations of both excipients, which could be explained by intermolecular protein interactions. Moreover, PS20 induced greater chemical-shift perturbations than PS80, demonstrating HOS modifications in the Fab region upon addition of PS20, suggesting that PS80 should be preferred in mAb formulations. As another example, the effect of silicone oil on the structure, stability, and aggregation of the dupilumab IgG4 therapeutic antibody was assessed by methyl 2D ^1^H-^13^C SOFAST HMQC on a 30 g/L solution of the mAb at natural abundance at 50 °C [59]. Overall, comparison of the methyl spectra of the mAb in the absence or presence of silicone oil showed that it does not induce significant modifications in the antibody structure.

The impact of formulation on the aggregation state of an antibody solution can also be monitored using pulsed field gradient NMR techniques, such as DOSY experiments [60]. However, while DOSY spectra allow straightforward differentiation between small molecules (e.g., 100 and 200 Da), distinguishing between large proteins of different sizes (e.g., 150 and 300 kDa) is much more difficult. Furthermore, DOSY is best suited for relative diffusion comparisons between molecules within a single sample, while obtaining absolute size values that can be reliably compared across different samples or different conditions is more challenging.

These examples illustrate the diversity of applications for methyl NMR fingerprinting and the range of analytical challenges for which this method is particularly valuable. The approach was also applied to compare Fc fragments from five marketed therapeutic IgG1s and one Fc-containing drug product, demonstrating that methyl fingerprints can characterize the HOS of a broad panel of therapeutics with minimal sample preparation [40]. This study aimed at developing a method to obtain high-resolution NMR information that could be easily applied to all IgG1 therapeutic mAbs.

However, 2D methyl spectra of mAb, Fab, and Fc recorded at natural isotopic abundance contain overlapped signals in the central spectral region, making visual detection of differences through direct spectral overlay more challenging. Manual interpretation becomes even more demanding when comparing a large number of spectra, underscoring the need for analysis tools capable of detecting statistically significant differences between methyl fingerprints.

### 3.5. Use of Principal Component Analysis to Compare Natural Abundance NMR Fingerprints

Given the strong potential of NMR fingerprinting to assess the HOS of monoclonal antibodies (mAbs), and its possible use as a quality-control tool during pharmaceutical development, the use of statistical approaches capable of comparing fingerprints and detecting HOS changes was evaluated. Such methods also help broaden the use of NMR fingerprinting to non-experts by enabling automated interpretation. Ideally, the tool should be able to classify NMR spectra of mAbs and their fragments and identify outliers that reflect significant structural modifications. Statistical tools could be implemented to compare 1D ^1^H spectra as well as 2D ^1^H-^15^N or ^1^H-^13^C methyl fingerprints.

Several approaches have been proposed to compare NMR spectra and detect outliers. Among them, the ECHOS method (Easy Comparability of HOS) provides a quantitative measure of HOS similarity between samples by comparing two 1D or 2D fingerprints. This similarity is derived from the correlation coefficient obtained through linear regression of the spectral matrices. When applied to ^1^H-^15^N SOFAST-HMQC spectra, the ECHOS method enabled clear differentiation between various antibody fragments [27]. As expected from their sequence similarity, the Fc fragments of the NISTmAb and a commercial polyclonal IgG1 (PolyAb) showed a high degree of spectral correlation. In contrast, a marked loss of correlation, reflecting noticeable spectral differences, was observed when comparing the Fab fragments of these two antibodies. The study further demonstrated that the ECHOS statistical framework can also be used to compare Fc fragment spectra before and after deglycosylation. ECHOS has also been applied to 1D ^1^H spectra of biologics [61], and more advanced approaches have recently emerged for comparing 1D NMR fingerprints of antibodies. Among them, the PROFOUND method, introduced by Elliott et al. [62], provides a robust framework for acquiring and analyzing 1D ^1^H spectra using NIPALS (Nonlinear Iterative Partial Least Squares) decomposition, with acquisition times suitable for routine use in mAb pharmaceutical development.

Principal component analysis (PCA) is another widely used multivariate technique for comparing 1D or 2D NMR spectra. It evaluates the variance across a large set of spectra and reduces the multidimensional NMR data into orthogonal variables known as principal components (PCs). PCA results are typically visualized using 2D score plots, which display the position of each spectrum along two principal components. These plots make it easy to observe clusters of similar samples, distinguish between sample groups, and identify potential outliers. For groups of replicates, 95% confidence ellipses are often added to the score plots. These ellipses represent the region where 95% of the points are expected to lie, assuming a normal distribution. They help assess whether groups of spectra are well separated, evaluate the variability among replicates, and highlight outliers.

Two main strategies exist for PCA of NMR data: using peak parameters such as chemical shifts, intensities, and line widths, or using the full spectral matrix [48]. Peak-based PCA provides a robust analysis independent of acquisition protocols or magnetic field strength, given that experimental sample conditions and spectral processing are standardized, thus being compatible with large studies involving several laboratories. However, it requires meticulous peak-picking for all spectra and is less sensitive to changes in crowded regions; also, new or missing peaks are only considered if manually identified beforehand. Conversely, PCA applied directly to the full spectral matrix allows fully automated comparison and detection of any spectral changes. This approach is particularly suitable when measurements are performed under rigorously controlled acquisition and processing conditions, using a single instrument and robust sample preparation, as small discrepancies such as baseline distortions, phase variations, or buffer signals can otherwise mask HOS-related spectral differences. PCA using spectral matrix data is then particularly suitable for industry, where very reproducible processes can be implemented [47,48].

PCA has been applied to a wide variety of NMR datasets, including 1D ^1^H spectra. For example, when used on sets of 1D ^1^H spectra from several biosimilars of infliximab and rituximab—two IgG1 antibodies with highly homologous sequences, superimposable Fab crystal structures, and identical Fc structures—PCA demonstrated its ability to reveal structural differences in enzymatically digested samples [63]. In this study, the PC1/PC2 score plots generated from the spectra of 15 rituximab and 7 infliximab samples clearly separated the two mAbs; their respective 95% confidence ellipses did not overlap, underlining the value of PCA for assessing the HOS of therapeutic antibodies. This study demonstrates that PCA of NMR spectra can serve as an effective analytical strategy for evaluating biosimilarity among antibodies, a capability of considerable interest for both health authorities and pharmaceutical companies.

Extensive research has been dedicated to evaluating PCA performance for detecting HOS variations in monoclonal antibodies using 2D ^1^H-^13^C NMR fingerprints. These studies have focused on selecting the appropriate PCA strategy based on context (e.g., peak lists versus full spectral matrices), optimizing key spectral processing parameters, and demonstrating PCA’s capability to discriminate between groups of spectra from mAbs or their fragments exhibiting distinct HOS features. Numerous investigations on the NISTmAb and its fragments—using enzymatically glycan-remodeled variants and oxidized samples—have produced practical guidelines for applying PCA to 2D NMR fingerprints of therapeutic mAbs in order to reveal HOS differences [38,45,47,48,52,64].

**Figure 11:**
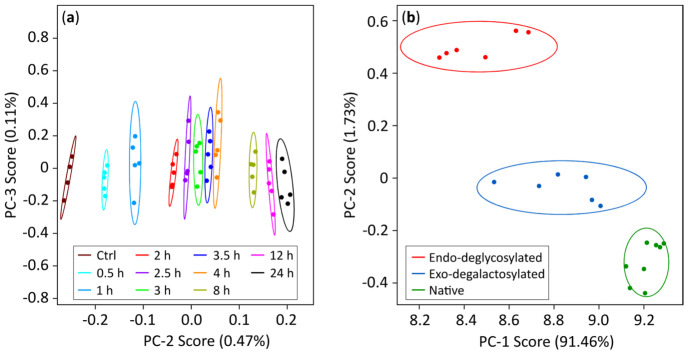
Examples of PCA score plots from ^1^H-^13^C methyl spectra of NISTmAb. (**a**) PCA score plot from 55 methyl spectra of NISTmAb at different time points after addition of hydrogen peroxide. Five replicates were analyzed for each time point. PC-1 represents the average spectrum, PC-2 extracts the spectral changes associated with oxidation, and PC-3 accounts for experimental variability and noise contributions. Adapted from [53]. (**b**) PCA score plot from 20 methyl spectra of NISTmAb isoforms (endo-deglycosylated, exo-degalactosylated, or native NISTmAb). Adapted from [45]. Ellipses correspond to the 95% confidence interval of the spread for each cluster.

PCA score plots derived from 2D spectra offer a highly intuitive visualization for classifying spectra based on the principal components that capture the dominant sources of variation within a dataset. By calculating 95% confidence ellipses for groups of spectra associated with specific sample conditions, one can assess both the significance of differences between groups and the variability within each condition. Score plots obtained from PCA of 2D ^1^H-^13^C NMR fingerprints of mAbs have proven effective for distinguishing between batches, glycoforms, and stressed samples, such as those subjected to oxidation or pH changes (Figure 11) [45,53,65].

Principal component analysis of 2D NMR fingerprints can provide more than the identification of significant differences between groups of spectra through score plots. Loading plots offer complementary insights by showing how strongly each original variable contributes to the principal components. When PCA is performed on full spectral matrices, the 2D loading plot associated with the principal component that captures the main variation between sample groups reveals the positions of NMR signals driving the observed differences in the fingerprints. These loading plots therefore support the interpretation of PCA outcomes by highlighting the spectral regions, or even individual resonances, that contribute most to the distinctions between samples.

To interpret these results accurately and extract detailed information about the observed changes in mAbs HOS, assigning each NMR signal to its corresponding methyl group is essential. Such assignments allow linking PCA-detected spectral variations to specific residues, thereby localizing structural modifications within the three-dimensional architecture of the protein. However, methyl resonance assignment of 50 kDa mAb fragments is technically demanding and requires high-quality heteronuclear 3D NMR experiments. Moreover, assigning methyl group resonances in proteins of this size typically requires prior assignment of backbone amide resonances, which in turn relies on triple-resonance experiments. Achieving the necessary sensitivity and resolution of NMR spectra depends on the use of samples isotopically enriched with ^2^H, ^13^C, and ^15^N. Over the past decades, several methodologies have been developed to produce labeled mAbs and antibody fragments suitable for such NMR studies, enabling the acquisition of high-resolution and high-sensitivity spectra that support robust signal assignment.

## 4. Overcoming a Major Challenge: Isotopic Labeling Strategies for NMR Investigations of Full-Length mAbs and Associated Fragments

Using monoclonal antibodies or their fragments at natural isotopic abundance has been shown to be suitable for acquiring NMR fingerprints. However, to extract atomic-resolution information about the HOS of therapeutic antibodies from such fingerprints, NMR signals must be assigned to their corresponding amino acids. Because recent studies have demonstrated that 2D ^1^H-^13^C methyl fingerprints are powerful tools for detecting changes in mAb HOS, considerable interest has focused on assigning methyl resonances in mAbs. As assignment becomes increasingly challenging with larger proteins, and because Fab and Fc NMR fingerprints overlap with those of the full mAb, fragments are generally studied individually rather than using the intact antibody. Further fragmentation strategies have recently been proposed to reduce the size of the antibody-derived proteins and thereby facilitate resonance assignment. Notably, the 25 kDa Single-chain Frament variable (ScFv), which comprises the V_L_ and the V_H_ domains linked together, has been used to assign the variable parts of IgG1 antibodies [49]. The use of isolated V_H_ and V_L_ domains to further simplify methyl group resonance assignment has also been investigated [66].

A commonly used strategy for methyl group resonance assignment involves first assigning backbone resonances through triple-resonance experiments, which edit ^1^H, ^13^C, and ^15^N frequencies. These assignments are then transferred to methyl side chains using 3D NMR experiments, often supplemented by NOESY-type experiments to complete and cross-validate the assignment. These experiments typically edit the ^1^H and ^13^C frequencies of methyl groups. Acquiring such 3D or 4D heteronuclear spectra requires isotopically enriched protein samples, with labeling schemes tailored to the specific NMR experiment. Isotopic enrichment plays two critical roles. First, enrichment in ^13^C and ^15^N increases their abundance by factors of approximately 90 and 280, respectively, thereby dramatically enhancing experimental sensitivity. Second, large proteins such as 50 kDa Fc and Fab fragments suffer from poor resolution because of rapid relaxation driven by dipole–dipole interactions between ^1^H nuclei. This effect can be mitigated by replacing non-essential protons with deuterium, reducing relaxation and improving spectral quality. Consequently, the optimal labeling scheme depends on both the protein and the type of NMR experiment and may require uniform or residue-specific enrichment with ^2^H, ^13^C, and ^15^N. For smaller fragments, typically up to about 30 kDa, uniform ^13^C and ^15^N labeling is generally sufficient. For larger proteins, however, partial or full deuteration becomes necessary to obtain high-quality spectra and enable reliable resonance assignment (Table 1).

For triple-resonance experiments, proteins are usually produced with uniform ^2^H, ^13^C, and ^15^N labeling, followed by proton-deuterium back-exchange at labile positions, including backbone amide sites, to enable ^1^H detection. Conversely, for smaller fragments such as ScFv or isolated mAb domains, fully protonated U-[^13^C,^15^N] samples may be suitable for backbone assignments. For experiments targeting methyl assignments (e.g., HCC-COSY, HCC-TOCSY or HMQC-NOESY-HMQC), specific labeling of methyl-containing residues is often required. Selective protonation of methyl groups within an otherwise fully deuterated protein can greatly reduce spectral crowding and enhance resolution. Likewise, selective ^13^C labeling of some methyl groups helps reduce overlap and facilitates resonance assignment.

Several expression systems can be used to produce isotopically labeled proteins, each offering distinct advantages and limitations. Selecting an appropriate system is particularly critical for mAb fragments, which contain multiple disulfide bonds and an essential N-glycosylation site at position N297, features that make their expression especially challenging. Considerable efforts have focused on developing methods to generate isotopically labeled mAbs or their fragments for NMR studies. Recent advances in producing labeled antibodies using CHO cells, *E. coli*, yeast, and cell-free expression platforms will be presented in this section.

### 4.1. Production of Isotopically Labeled Full-Length mAbs in CHO Cells

In the pharmaceutical industry, therapeutic mAbs are typically produced by cultivating CHO cells in optimized, nutrient-rich media, enabling very high antibody yields per liter of culture. CHO cells naturally provide all expected post-translational modifications, including the conserved N297 glycosylation site in the Fc fragment. Producing labeled mAbs in CHO cells therefore offers a major advantage: it yields intact antibodies comparable to industrial therapeutic mAbs, which can subsequently be digested to obtain isotopically labeled fragments for NMR studies.

Several studies have reported the production of uniformly ^13^C- and/or ^15^N-labeled IgG1 antibodies [67]. Antibody-producing CHO cell lines can be grown in a serum-free synthetic medium supplemented with amino acids, glucose, and carboxylic acids as metabolic precursors. Uniform isotopic labeling is achieved by substituting these precursors with their labeled equivalents. However, individually supplying all 20 labeled amino acids is particularly expensive. As a cost-saving alternative, labeled amino acids are often provided as hydrolyzed algal extracts grown with inexpensive labeled precursors. These mixtures must still be supplemented with isotope-labeled amino acids degraded during acid hydrolysis. Using this strategy, U-[^13^C,^15^N] IgG1 was successfully produced in a modified Nissui NYSF 404 medium in which glucose, sodium pyruvate, succinic acid, and amino acids were replaced by D-[^13^C_6_] glucose, [^13^C_3_] pyruvic acid, [^13^C_4_] succinic acid, and a [^13^C, ^15^N] algal amino acid mixture supplemented with [^13^C_6_,^15^N_4_] L-arginine, [^13^C_4_,^15^N_2_] L-asparagine, [^13^C_3_,^15^N] L-cysteine, [^13^C_5_,^15^N_2_] L-glutamine, [^13^C_6_,^15^N_3_] L-histidine, [^13^C_9_,^15^N] L-tyrosine, and [^13^C_11_,^15^N_2_] L-tryptophan. The glycosylated Fc fragment was then cleaved with papain and purified for NMR characterization [67,68,69,70,71]. A major cost driver in uniform labeling is isotope-labeled glutamine, required in high concentrations because of its essential role in energy metabolism as well as nucleotide and amino acid biosynthesis. To reduce costs, Yanaka et al. proposed a strategy in which cells are cultured in a glutamine-free medium, using a glutamine synthetase capable of synthesizing glutamine from glutamate and ammonium. This approach enabled the production of 50 mg of U-[^15^N] rituximab per liter of culture, with significant cost savings of approximately 50% for the NMR sample preparation [72,73].

Selective ^13^C, ^15^N amino acid labeling is also feasible in CHO cells by supplementing the culture medium with individually labeled amino acids [67,68,74]. More recently, a strategy enabling the production of antibodies containing specifically enriched in ^2^H- and ^13^CH_3_-labeled amino acids was described [75]. Using an amino-acid-depleted medium supplemented with the 20 amino acids, each with the desired labeling scheme, the authors produced an IgG1 containing deuterated methyl-containing amino acids, and labeled with ^13^CH_3_ on alanines-β, isoleucines-δ_1_, methionines-ε, leucines-δ_2_, threonines-γ, and valines-γ_1_, achieving a favorable yield of 80 mg/L. This labeling is regioselective for isoleucines and stereoselective for leucines and valines, ensuring that only one methyl group per methyl-containing amino acid is labeled, thereby reducing the number of signals and limiting potential signal overlaps. This strategy enabled high-quality ^1^H-^13^C NMR spectra of the full IgG1, where almost all expected methyl signals were well resolved. A similar approach was used to produce rituximab samples in which a single methyl-containing amino acid was isotopically labeled [76]. Amino acids with diverse labeling patterns were successfully incorporated, including U-[^13^C,^15^N] threonine or isoleucine; valine or leucine stereospecifically labeled with ^13^C at the γ_1_ or δ_2_ methyl position, respectively; and valine or leucine labeled at one or the other methyl group without stereospecificity.

Interestingly, the CHO cell expression system also allows isotopic labeling of glycans. For instance, replacing unlabeled D-glucose with [^13^C_6_] D-glucose results in ^13^C enrichment of carbohydrate carbons, enabling direct NMR observation of glycan signals [67,74]. Furthermore, ^13^C and ^15^N labeling of the acetamide groups of N-acetylglucosamine residues can be achieved by supplementing the medium with [^13^C_6_] D-glucose and [^15^N] glucosamine [77].

Despite these advantages, CHO cells are not suitable for producing fully deuterated proteins, due to the cytotoxicity of D_2_O. As extensive deuteration is essential for comprehensive side-chain methyl resonance assignment of mAb fragments, alternative expression systems must therefore be considered. It is worth noting that protein production in CHO cells remains a costly and time-intensive process.

### 4.2. Expression of Labeled Antibody Fragments in E. coli

*E. coli* bacteria are widely used for protein overexpression due to their low cultivation cost and the high yields they typically provide. Because they are also known to support a high level of deuteration, proteins labeled with ^2^H, ^13^C, and ^15^N for NMR studies are mainly produced in *E. coli*. A common strategy consists of growing the cells in minimal medium supplemented with labeled glucose and labeled ammonium as the sole carbon and nitrogen sources. Production can be carried out in H_2_O or D_2_O to obtain protonated or perdeuterated proteins. Overall, numerous labeling strategies have been developed to optimize spectral quality and enable reliable methyl assignments [78]. However, the production of glycosylated monoclonal antibodies is not possible in *E. coli*, since this bacterial expression system does not support the post-translational modifications required for glycan processing. It is nevertheless possible to produce non-glycosylated mAbs or mAb fragments in *E. coli*. However, as these proteins contain multiple polypeptide chains and numerous disulfide bonds, obtaining them properly folded is particularly challenging. Several strategies have been developed to express isotopically labeled and correctly folded antibody fragments in *E. coli*.

A commonly used approach consists of expressing the fragments as inclusion bodies followed by in vitro refolding. To produce heterodimer fragments of antibodies, the expression of a single polypeptide chain containing the two chains attached together with a linker was proposed to facilitate refolding from expression in inclusion bodies [49,79]. For instance, a single-chain Fab (scFab) construct consists of the light chain attached to the Fab part of the heavy chain, and a single-chain Fragment variable (ScFv) construct corresponds to the V_L_ and the V_H_ sequences linked together. This *E. coli* expression strategy enabled the production of trastuzumab Fab under three different labeling schemes: U-[^2^H,^13^C,^15^N], U-[^2^H,^13^C,^15^N]-^1^H-methyl-[I,V,L], and U-[10% ^13^C,^15^N]. For this purpose, *E. coli* BL21(DE3) cells carrying the scFab gene were grown in minimal M9 medium supplemented with labeled glucose and labeled ammonium [79]. For the U-[^2^H,^13^C,^15^N] condition, the culture was performed in M9/D_2_O supplemented with [^2^H, ^13^C] glucose and ^15^N ammonium chloride as the sole carbon and nitrogen sources. The same strategy was used to produce the U-[^2^H,^13^C,^15^N]-^1^H-methyl-[I,V,L] scFab, with the addition of U-[^13^C]-3,3-d_2_-α-ketobutyric acid and U-[^13^C]-3-d_1_-α-ketoisovaleric acid during the culture. The U-[10% ^13^C,^15^N] labeling condition, used for stereospecific assignments of leucine and valine methyl groups, was obtained by growing *E. coli* in M9/H_2_O supplemented with 10% of ^13^C-glucose, 90% of unlabeled glucose, and ^15^N-ammonium chloride. After fast-dilution refolding, cleavage of the linker between the heavy and light chains, and purification of the scFab, an average yield of 40 mg per liter of culture was achieved [79,80]. A very similar protocol was employed for producing the scFab fragment of adalimumab labeled as follows: U-[^2^H,^13^C,^15^N] and U-[^2^H,^13^C,^15^N]-^1^H-methyl-[I,V,L] [81]. Recently, an innovative approach was developed in which the light and heavy chains of Fab fragments are produced separately as inclusion bodies and subsequently refolded together to generate the correctly folded fragment. This strategy enables differential isotopic labeling of the two chains. Using this method, yields of up to 40 mg of Fab per liter of culture were obtained, with one chain uniformly or specifically labeled while the other remained at natural isotopic abundance [82]. Moreover, the strategy of protein expression in inclusion bodies followed by a refolding step also enabled the production of U-[^15^N,^13^C] and U-[^15^N] labeled NISTmAb ScFv fragments [49], as well as a non-glycosylated IgG1 Fc fragment labeled U-[^2^H,^13^C,^15^N] [83]. Because refolding is performed in a protonated buffer, deuterium atoms at exchangeable sites are replaced by protons during this step, allowing the production of perdeuterated fragments with protonated amide groups, an essential requirement for many NMR experiments used in backbone resonance assignment.

Cha et al. proposed another strategy that avoids refolding by producing a U-[^13^C,^15^N] Fab directly in the periplasm of *E. coli*. To improve expression yields in the periplasm, induction was carried out at OD_600_ = 1.5 instead of the conventional 0.6. After purification, the correctly folded labeled Fab was obtained with a yield of approximately 5 mg per liter of culture [84]. However, this method is not suitable for producing perdeuterated fragments with protonated amides, since full unfolding is required to ensure complete back-protonation of buried NH groups.

Finally, a recently reported study demonstrated the feasibility of using an *E. coli* strain, Shuffle T7, genetically engineered to express the disulfide bond isomerase DsbC, enabling disulfide-bond formation in the cytoplasm to produce labeled antibodies. Using this strain, the full NISTmAb singly labeled with ^13^C or ^15^N, or triply labeled with ^2^H, ^13^C, and ^15^N was expressed, and correctly folded purified mAb was obtained at approximately 1 mg per liter of culture, without requiring a refolding step [51].

In summary, *E. coli* offers a straightforward and cost-effective platform for producing isotopically enriched mAbs and their fragments, supporting a broad variety of labeling strategies. Although these proteins lack glycosylation, the 2D NMR spectra of mAb fragments expressed in *E. coli* closely match those obtained from CHO-derived mAbs. However, achieving correctly folded fragments typically requires an extensive refolding process, which can significantly reduce yields. In addition, implementing more sophisticated labeling approaches may be challenging due to isotopic scrambling that can occur during protein synthesis.

### 4.3. Production of Isotopically Enriched Fab in Pichia pastoris Yeast

*Pichia pastoris* yeast has also been investigated as an expression system for producing labeled mAb fragments. Like *E. coli*, *P. pastoris* can grow on simple nitrogen and carbon sources that can be replaced with their labeled counterparts, and it tolerates high levels of D_2_O in culture media. Furthermore, *P. pastoris* can produce N-glycosylated Fc fragments, although the N-glycans differ from those found in humans. This yeast is also well suited for expressing multi-chain soluble proteins rich in disulfide bonds and can directly secrete properly folded proteins into the growth medium.

This approach was used to produce NISTmAb Fab fragments with various labeling schemes. *P. pastoris* grown in minimal media supplemented with ^15^N ammonium sulfate and ^13^C methanol yielded U-[20% ^13^C,^15^N]-labeled Fab fragment and U-[^13^C,^15^N] labeled Fab fragment with the addition of ^13^C glucose with production levels of 2 to 6 mg of purified Fab per liter of culture [79,85]. Overlaying the NMR spectrum of the yeast-produced NISTmAb Fab with that of the reference NISTmAb Fab showed that nearly all resonances align closely, indicating highly similar 3D structures. However, when switching to doubly labeled ^2^H, ^15^N Fab using glucose as the sole carbon source in D_2_O, the production yield decreased significantly due to the difficulty of achieving high cell density under these conditions [79]. Consequently, producing triply labeled Fab using doubly labeled glucose as the only carbon source would be prohibitively expensive. To address this, a triply labeled rich medium was used, incorporating ^2^H, ^13^C, ^15^N ISOGRO^®^ into minimal media [54]. However, to fully exchange amide deuterons back to protons, a partial unfolding step was required.

Overall, *P. pastoris* offers the advantage of secreting well-folded mAb fragments into the culture medium and tolerating high levels of deuteration. However, cost-effective strategies for generating deuterated fragments lead to poor production yields, and refolding remains necessary to achieve full amide protonation needed for NMR signal recovery.

### 4.4. E. coli-Based Cell-Free System for mAb Fragment Production with Unlimited Labeling Schemes

Recently, cell-free expression systems based on *E. coli* extracts have emerged as a promising solution for producing isotopically labeled mAb fragments. Although they do not enable N-glycosylation, cell-free systems offer several advantages, including rapid expression, broad labeling possibilities, and the absence of refolding steps. Because transcription and translation occur in an open, membrane-free environment, any molecule that enhances protein expression or stability, such as cofactors or chaperons, can be directly added to the reaction mixture. For example, purified DsbC enzyme can be supplemented to promote correct folding of mAb fragments during cell-free expression. Furthermore, since the translation machinery incorporates the amino acids supplied in the reaction, substituting them with isotopically labeled counterparts allows a wide range of labeling strategies. The 20 amino acids can be added individually with defined labeling patterns, provided that they are commercially available. It is therefore possible to produce a mAb fragment in which all amino acids are deuterated while keeping protonated amide groups, without requiring any refolding, by performing the reaction in H_2_O with deuterated amino acids [86]. Conversely, the reaction can also be performed in D_2_O with deuterated substrates and selectively protonated amino acids at specific sites to obtain a perdeuterated protein with specific protonation. Furthermore, methyl-specific isotopic enrichment can be achieved by incorporating one or several ^13^CH_3_-labeled amino acids in the reaction mixture. All labeling schemes required for backbone and methyl-group resonance assignments can thus be achieved using cell-free expression.

This approach enabled the production of correctly folded Fab targeting lysosomal-associated membrane protein 1 (LAMP1), through co-expression of the light and heavy chains. At natural abundance, up to one milligram of purified Fab was obtained from one milliliter of reaction mixture after optimization. U-[^2^H,^13^C,^15^N] anti-LAMP1 Fab with protonated amide groups was produced by supplying the twenty U-[^2^H,^13^C,^15^N] amino acids in a protonated reaction mixture, allowing backbone resonance assignment of the Fab fragment [87]. To reduce production costs, uniform labeling can also be achieved using labeled algal extracts supplemented with cysteine and tryptophan. Using this approach, U-[^13^C,^15^N] scFv, V_L_, and IgG1 Fc fragments were produced with reasonable yield-to-cost ratios [66,88]. Methyl-specific labeling of mAb fragments has also been achieved using *E. coli*-based cell-free systems. For instance, a U-[^2^H] Fab sample with ^13^CH_3_ labeling on alanines-β, isoleucines-δ_1_, methionines-ε, leucines-δ_2_, threonines-γ, and valines-γ_1_, as well as a U-[^2^H] Fab sample with U-[^13^C]- and ^13^CH_3_-labeled alanines-β, isoleucines-δ_1_, and valines-γ_1_, were produced using fully deuterated reaction mixtures supplemented with specifically labeled amino acids [66]. Overall, because any desired labeling scheme can be implemented in an *E. coli*-based cell-free system, limited only by commercial availability of amino acids or precursors, sample preparation can be fully tailored to the NMR experiments of interest. In particular, amino-acid-specific, regiospecific, and stereospecific methyl labeling is highly valuable for methyl group resonance assignment.

To conclude, all expression systems present distinct advantages and limitations for the production of isotopically enriched mAb fragments (Table 2). The choice of production platform should consider the type of mAb fragment required (especially regarding glycosylation), the desired labeling scheme, and the accessibility of the method. Although *E. coli* is typically the most accessible option, it often requires complex and labor-intensive refolding steps. Yeast offers an attractive alternative because it can secrete folded fragments directly into the culture medium, but yields drop sharply when high levels of deuteration are required. In contrast, *E. coli*-based cell-free expression systems provide extensive flexibility in labeling schemes and can be readily optimized to improve the yield and stability of antibody fragments. Furthermore, protein expression in cell-free systems requires substantially less volume of medium than the other techniques described for producing an equivalent amount of protein. This is particularly advantageous when working with very expensive D_2_O-based media. Moreover, the high incorporation yield of isotopes enables the use of limited quantities of isotopically labeled material, compensating for the important cost of ^13^CH_3_-labeled and perdeuterated amino acids [89]. In addition, cell-free protein expression is considerably faster than more conventional expression systems. CHO cells offer the advantage of producing glycosylated antibodies under similar conditions to those used for therapeutic mAbs; however, perdeuteration cannot be achieved, and the production process is considerably longer and more demanding. Less conventional expression systems, such as silkworms, transgenic tobacco, and mouse hybridoma cell lines, have also been evaluated for the production of ^13^C- and ^15^N-enriched mAbs and fragments, but they offer no remarkable advantages compared to the more established approaches described above [90,91,92,93,94,95]. Although no expression system is perfectly adapted to the production of ^2^H-, ^13^C-, and ^15^N-enriched antibody samples, the recent advances in recombinant expression of mAb fragments and isotopic labeling have paved the way for the acquisition of high-quality NMR spectra using labeled samples, therefore enabling resonance assignment work.

## 5. Resonance Assignment: Towards HOS Characterization at Atomic Resolution

The development of innovative strategies for producing isotopically enriched antibody fragments has enabled several research groups to record high-quality NMR spectra on uniformly or selectively labeled antibody fragment samples, thereby opening the doors to comprehensive NMR assignment. A widely used strategy for assigning methyl group resonances involves first assigning backbone resonances and then transferring these assignments to methyl groups using 3D NMR experiments and specifically labeled samples.

### 5.1. Backbone Assignment of Antibody Fragments

**Figure 12:**
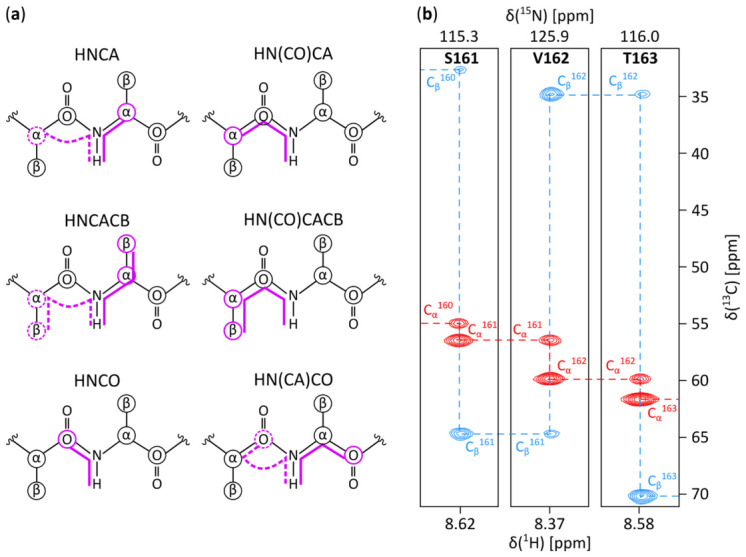
Backbone sequential assignment. (**a**) Schemes representing the transfer of magnetic resonance occurring during HNCA, HN(CO)CA, HNCACB, HN(CO)CACB, HNCO, and HN(CA)CO triple-resonance experiments. Transfer is represented by purple lines. For HNCA, HNCACB, and HN(CA)CO, the magnetic transfer passing through the C^α^ of the i-1 residue is represented by dashed lines. For each experiment, carbon atoms for which a signal is expected are circled in purple. (**b**) 2D extracts from HNCA and HNCACB spectra acquired on a U-[^2^H,^13^C,^15^N] sample of anti-LAMP1’s Fab. The dotted lines represent the sequential assignment of residues S161, V162, and T163. C^α^ resonances are depicted in red and C^β^ resonances in blue. HNCA and HNCACB spectra were acquired at 35 °C on a 950 MHz spectrometer equipped with a cryogenic probe, using a 0.3 mM sample.

To assign backbone resonances of 50 kDa proteins, uniformly ^2^H-, ^13^C-, and ^15^N-labeled samples with protonated amides are typically required. A set of triple-resonance experiments, namely HNCA, HN(CO)CA, HNCACB, HN(CO)CACB, HNCO, and HN(CA)CO, can then be acquired and analyzed for sequential backbone assignment (Figure 12) [96]. This strategy enables the assignment of H^N^, N, C’, C^α^, and C^β^ resonances of all amino acids, except for H^N^ and N resonances of proline residues.

The first published backbone assignment of an antibody fragment corresponds to the variable fragment (Fv) of an IgG2a antibody, which is a heterodimer comprising the V_L_ and V_H_ domains [97]. No deuteration was used for this 25 kDa protein, but several samples with uniform or residue-specific ^13^C and ^15^N labeling were required, yielding a near-complete assignment of H^N^, N, and C^α^ resonances.

The Fc fragment of IgG1 antibodies has since been extensively studied, resulting in multiple backbone assignments (Table 3). An initial 66% of NH groups were assigned on several glycoforms (G2F, G0F, M3F, FGN and deglycosylated) of an IgG1 Fc produced in CHO cells and obtained after successive glycan trimmings [67]. The assignment of the G0F glycoform of an IgG1 Fc was later completed using a combination of uniformly and amino-acid-selectively ^13^C- and ^15^N-labeled samples, achieving 99% assignment of H^N^, N, and C^α^, 84% of C^β^, and 80% of C’ resonances [68]. These assignment percentages are remarkably good, especially considering that they were achieved for a 50 kDa homodimer without deuteration. This work enabled the assignment of the G0, G2, and G2F glycoforms of IgG1 Fc [72,77]. Backbone assignments were also reported for the G0 and G2 glycoforms of an IgG2b Fc [71]. Notably, all these assignments of glycosylated Fc fragments were performed using antibodies produced in CHO cells, and thus without deuteration. Backbone resonances of the non-glycosylated IgG1 Fc fragment were also successfully assigned using a U-[^2^H,^13^C,^15^N]-labeled sample produced in *E. coli*, resulting in 90% assignment of H^N^, N, C’, C^α^, and C^β^ resonances for non-proline residues [83]. Because the Fc fragment is highly conserved among antibodies of the same IgG subtype, these assignments are broadly applicable for studying most therapeutic mAbs.

In contrast, the Fab fragment is dependent on the antibody target, meaning that the assignment process must be performed for each antibody. Since NMR spectra of Fab fragments contain roughly twice as many signals as Fc fragments, their assignments are correspondingly more complex. Since 2022, a few backbone assignments have been reported for four full-length Fabs and three smaller constructs (Table 3). Assignments of the smaller constructs—namely the single-chain variable fragments of an anti-MSP2 mAb (targeting the malaria antigen merozoite surface protein 2) [98], the NISTmAb [49] and ipilimumab [66], as well as the V_L_ domain of ipilimumab [66]—were obtained using uniformly ^13^C, ^15^N-labeled samples. For the larger 50 kDa Fab fragments, perdeuteration becomes essential. Production of U-[^2^H,^13^C,^15^N]-labeled samples in *E. coli*, yeast, or cell-free expression systems enabled successful backbone assignments of the Fabs from NISTmAb [54], trastuzumab [80], anti-LAMP1 [87], and adalimumab [81].

**Table 3:** List of the BMRB deposition numbers corresponding to backbone assignments of mAb fragments.

BMRB Number	Assigned Fragment	Assignment Percentage ^1^	Publication Year
4580 [97]	Fv (IgG2a, anti-dansyl)	98%	2000
15514 [83]	Fc non-glycosylated (IgG1)	95%	2007
15204 [99]	CH3 domain (IgG1)	100%	2007
25224 [68]	Fc G0F (IgG1)	99%	2015
50515 [71]	Fc G0 (IgG2b)	97%	2021
50522 [71]	Fc G2 (IgG2b)	97%	2021
51059 [72]	Fc G0 (IgG1)	90%	2022
51094 [49]	scFv (IgG1, NISTmAb)	87%	2022
51696 [54]	Fab (IgG1, NISTmAb)	94%	2023
52228 [80]	Sc-Fab (IgG1, trastuzumab)	88%	2024
52243 [87]	Fab (IgG1, anti-LAMP1)	89%	2024
52274 [81]	Fab (IgG1, adalimumab)	91%	2024
52917 [77]	Fc G0F (IgG1)	83%	2025
52918 [77]	Fc G2F (IgG1)	82%	2025
52919 [77]	Fc G2 (IgG1)	89%	2025
52921 [77]	Fc G0 (IgG1)	82%	2025
53197 [82]	Fc non-glycosylated (IgG1-EEM)	90%	2026
53199 [82]	Fc non-glycosylated (IgG1-DEL)	91%	2026
53420 [66]	V_L_ (IgG1, ipilimumab)	60%	2026
53421 [66]	ScFv (IgG1, ipilimumab)	52%	2026
53440 [82]	Fab (IgG1, bevacizumab)	91%	2026
53491 [82]	Fab (IgG1, rituximab)	95%	2026
53625 [82]	Fc G0, G1, G2 (IgG1-EEM)	90%	2026

^1^ The assignment percentages were calculated as the number of available H^N^ and N resonances in the BMRB files divided by the total number of amino acids, excluding prolines.

These numerous assignments of both Fab and Fc fragments provide residue-level structural information from standard 2D ^1^H-^15^N-correlated NMR spectra. They can therefore be used either to detect differences between antibody batches or to localize changes induced by stress conditions. For instance, the backbone assignment of the scFv construct of an anti-MSP2 mAb was used to localize interactions between the scFv and its antigen by plotting chemical shift perturbations versus residue number, based on spectra acquired on the labeled scFv in the absence or presence of the antigen [98]. Such use of the backbone assignment can also be considered with unlabeled material. As an example, Hodgson and co-workers used the backbone resonance assignment of the Fc fragment of an IgG1 [68] to compare 2D ^1^H-^15^N-correlation spectra of the glycosylated Fc fragment and the Endo-S treated Fc of rituximab at natural abundance. This assignment enabled the mapping of chemical shift perturbations onto the structure, providing precise structural insights into the Fc region upon glycan cleavage. However, each spectrum required 108 h of acquisition to achieve sufficient sensitivity, making this approach unsuitable for routine quality control analysis of therapeutic mAbs. This limited sensitivity arises from both the low natural abundance of the ^15^N isotope (0.36%) and its relatively low gyromagnetic ratio, making ^1^H-^13^C methyl spectra better fingerprints than ^1^H-^15^N ones. In addition, ^1^H-^13^C methyl fingerprints present fewer and better resolved signals than ^1^H-^15^N ones. In the context of HOS characterization of mAbs during therapeutic development, backbone assignments are therefore generally considered more as a means to enable methyl-group assignment than as an end goal in themselves.

### 5.2. Assignment of Side-Chain Methyl Resonances in mAb Fragments

**Figure 13:**
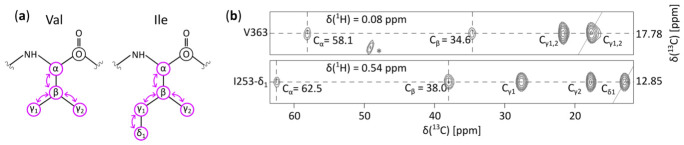
Assignment transfer strategy from backbone to methyl groups using TOCSY experiments. (**a**) Scheme of the magnetization transfer (represented with purple arrows) occurring during TOCSY mixing time in valine and isoleucine residues. (**b**) 2D extracts from an HCCH-TOCSY experiment acquired on a U-[^13^C,^15^N] non-glycosylated Fc sample. 2D extracts are displayed correlating previously assigned C^α^ and C^β^ resonances to V363 and I253 methyl resonances. The asterisk indicates a signal outside of the considered plane. Adapted from [88].

Once backbone resonance assignments are available, the first step in assigning side-chain methyl resonances of proteins typically involves transferring the C^α^ and C^β^ assignments to the side-chain carbon and proton resonances using TOtal Correlation SpectroscopY (TOCSY) or COrrelation SpectroscopY (COSY) experiments. In HCCH-TOCSY experiments, magnetization is transferred from side-chain ^1^H to the attached ^13^C, then dispersed to other side-chain ^13^C through isotropic ^13^C mixing, and finally transferred back to ^1^H for detection. In HCCH-COSY, by contrast, magnetization is exchanged between neighboring ^13^C nuclei via J-couplings rather than isotropic mixing. Both approaches aim to correlate methyl-group resonances with other side-chain carbons and protons, including C^α^ and C^β^, enabling propagation of the backbone assignment to the methyl groups (Figure 13). These experiments can be performed on protonated U-[^13^C] samples for small proteins, and require perdeuteration, linearized ^13^C labeling as well as specific protonation at the methyl positions for proteins larger than 50 kDa [66,88,100].

For small antibody fragments, such as V_L_ and C_H_3 domains, scFv, and the 50 kDa Fc dimer, uniform ^13^C, ^15^N labeling is sufficient to acquire HCCH-TOCSY and HCCH-COSY spectra and transfer the assignment from the backbone to the methyl groups [49,66,88,99]. For Fab fragments, which are 50 kDa heterodimers, deuteration becomes necessary as ^1^H dipole–dipole relaxation leads to decreased signal resolution. Two strategies have been proposed for transferring backbone assignments to methyl groups. The first, applied by Aubin and co-workers on Fab fragments, uses [U-[^2^H,^13^C,^15^N],^1^H-Ile^δ1^,^1^H-Leu^δ1,δ2^, ^1^H-Val^γ1,γ2^] single-chain Fab with protonated NHs, combined with a set of 3D TOCSY-based experiments (CCC(CO)NH, HCC(CO)NH, CCC(CA)NH, HCC(CA)NH) to link the methyl ^1^H and ^13^C resonances of Ile^δ1^, Leu, and Val to assigned H^N^ and N backbone signals [80,81,101]. The second strategy employs a U-[^2^H] Fab sample with selective ^13^CH_3_-labeling on alanine-β, isoleucine-δ_1_, and valine-γ_1_ and a linearized chain of ^13^C, enabling acquisition of 3D HCC, HC(C)C, and HC(CC)C COSY experiments, connecting methyl resonances to assigned C^α^ and C^β^ resonances [66].

Although TOCSY- and COSY-based strategies are very useful for transferring assignments from the backbone to methyl groups, they are generally not sufficient to complete methyl resonance assignment. Many residues share similar C^α^ and/or C^β^ chemical shifts, which may lead to ambiguous assignments when spectral resolution is insufficient to distinguish them. Moreover, when protein deuteration is required, the sophisticated labeling schemes needed for these experiments can be challenging to implement, and achieving the appropriate labeling pattern may be particularly difficult for certain methyl-bearing amino acids. Notably, deuterated threonine labeled with ^13^C on the side chain and selectively protonated only on the methyl group is not commercially available. Moreover, this strategy is not adapted to methionine residues, as the sulfur atom limits magnetic transfer between the methyl carbon and the other carbons of the side chain. As a result, backbone-to-methyl transfer should be regarded as an initial step in the methyl-assignment process that typically requires complementary experiments to fully and unambiguously complete the assignment.

**Figure 14:**
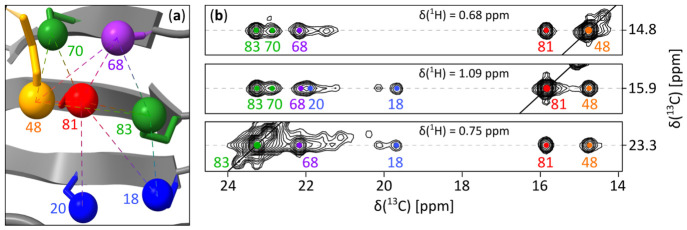
Methyl group assignment strategy using a NOESY experiment. (**a**) 3D structure of anti-LAMP1 Fab, zoom in on the variable part of the heavy chain (PDB 8ATH) [102]. Alanines-β (purple), isoleucines-δ_1_ (orange), valines-γ_1_ (blue), leucines-δ_2_ (green), and methionines-ε (red) are represented by spheres. Dashed lines are drawn between methyl groups when a NOE cross-peak is observed for residues 48, 81, and 83. (**b**) 2D extracts from a 3D HMQC-NOESY-HMQC using an anti-LAMP1’s Fab sample U-[^2^H] and specifically labeled on Ala-[^13^CH_3_]^β^, Met-[^13^CH_3_]^ε^, Leu-[^13^CH_3_]^δ2^, Val-[^13^CH_3_]^γ1^, Ile-[^13^CH_3_]^δ1^, and Thr-[^13^CH_3_]^γ^. Planes were extracted at the methyl proton frequencies of I48, M81, and L83. Adapted from [66].

To further complete methyl-group resonance assignments, NOESY (Nuclear Overhauser Effect SpectroscopY) experiments are generally employed. In these experiments, magnetization is transferred between protons that are spatially close, enabling detection of through-space correlations. When combined with an initial set of assignments and a 3D structure of the protein, NOESY data can provide valuable constraints for assigning methyl resonances. For instance, ^13^C- and ^15^N-edited NOESY-HSQC experiments have been used to complete and validate the assignments of both the C_H_3 domain of an IgG1 [99] and the NISTmAb scFv fragment [49], using uniformly ^13^C, ^15^N-labeled samples. Another particularly useful experiment is the CCH HMQC-NOESY-HMQC, which has proven effective for completing and validating methyl assignments in both the anti-LAMP1 Fab fragment and the non-glycosylated Fc fragment of an IgG1 [66,88]. In these 3D spectra, each methyl signal displays a band of NOE cross-peaks corresponding to nearby methyl groups, providing spatial information that supports the assignment process (Figure 14).

**Figure 15:**
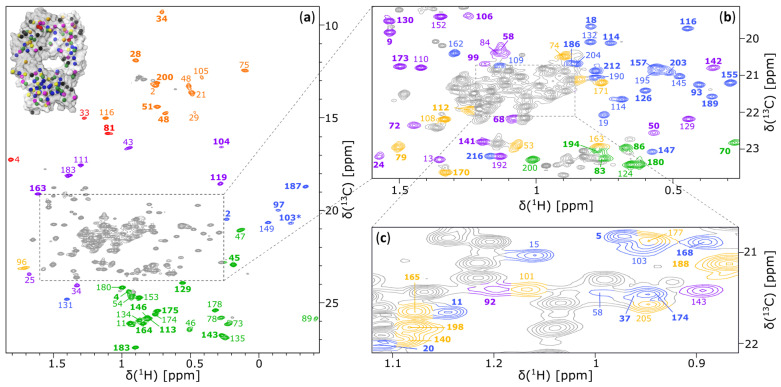
Assigned 2D ^1^H-^13^C methyl spectrum of the Fab fragment of anti-LAMP1 mAb. The Fab fragment was produced in a cell-free system and U-[^2^H], Ala-[^13^CH_3_]^β^, Met-[^13^CH_3_]^ε^, Leu-[^13^CH_3_]^δ2^, Val-[^13^CH_3_]^γ1^, Ile-[^13^CH_3_]^δ1^, Thr-[^13^CH_3_]^γ^ specifically labeled. Alanines are depicted in purple, isoleucines in orange, methionines in red, leucines in green, threonines in yellow, and valines in blue. In bold are resonances belonging to the heavy chain and in regular are the ones belonging to the light chain. The asterisk indicates that the contour level of the residue 104 has been multiplied by 3. Signals depicted in gray are either impurities, residues from the tags or unassigned signals. Most of the unassigned signals correspond to threonines, as only 40% of threonine methyl signals could be assigned. (**a**) Full spectrum. Top left, crystal structure of the Fab fragment, methyl groups are represented by spheres and are colored by amino acid type if assigned, and in black otherwise. (**b**) Zoom from panel (**a**). (**c**) Zoom from panel (**b**), contour level has been decreased by 2. Adapted from [66].

For large proteins such as Fc or Fab fragments, these experiments must be performed on specifically labeled samples. In the case of the Fab fragment of anti-LAMP1, a U-[^2^H,^12^C], Ala-[^13^CH_3_]^β^, Ile-[^13^CH_3_]^δ1^, Val-[^13^CH_3_]^γ1^, Thr-[^13^CH_3_]^γ^, Met-[^13^CH_3_]^ε^, Leu-[^13^CH_3_]^δ2^ labeling scheme was used. For the Fc fragment, a combination of (i) Ala-[^13^CH_3_]^β^, Ile-[U-[^2^H], [^13^CH_3_]^δ1^], Met-[^13^CH_3_]^ε^, Thr-[U-[^2^H], [^13^CH_3_]^γ^], and Val-[U-[^2^H], [^13^CH_3_]^γ1/γ2^]; (ii) Leu-[U-[^13^C], [^13^CH_3_]^δ1,δ2^]; and (iii) Val-[U-[^2^H], [^13^CH_3_]^γ1,γ2^] labeling patterns was employed. These tailored labeling schemes enabled near-complete assignment of the methyl resonances for both Fab and Fc fragments of an IgG1 (Figure 15).

For isoleucine, leucine, and valine residues, which each contain two methyl groups, the assignment depends primarily on the chosen labeling strategy. When non-stereospecifically labeled amino acids are used, both methyl groups can typically be assigned to the corresponding residue, but without stereospecific distinction between the two positions [49,66,88,99]. In contrast, when only one methyl group per residue is desired, stereospecific labeling schemes can be employed, using precursors or amino acids to selectively label a single methyl site of valine, isoleucine, or leucine during protein expression [66,76,103]. Finally, stereospecific assignment of the two methyl groups of valine and leucine residues can be achieved by recording a 2D ^1^H-^13^C Constant-Time-HSQC (CT-HSQC) spectrum on a protein expressed in cells grown in minimal medium containing 10% [^13^C_6_]-glucose and 90% natural-abundance glucose as the sole carbon source. For valine biosynthesis, the γ_1_ and β carbons originate from the same pyruvate molecule, whereas the γ_2_ carbon is derived from a second pyruvate molecule. As a result, approximately 10% of valines are labeled with ^13^C at γ_1_ and β, another 10% at γ_2_ with ^12^C at β, and roughly 1% contains ^13^C at β, γ_1_, and γ_2_ positions simultaneously. Because the phase of a methyl signal in a CT-HSQC experiment depends on the number of directly attached ^13^C nuclei, the γ_1_ and γ_2_ methyl groups exhibit opposite signs under these labeling conditions [104,105]. A similar labeling pattern occurs for leucines, allowing stereospecific assignment of both methyl groups of valines and leucines using the same sample and a single 2D experiment [80].

The assignment of the glycosylated Fc fragment of IgG1 rituximab was performed by Yanaka and coworkers using samples of the Fc labeled at only a single methyl-containing amino acid [76]. Similar to the approaches described above, assignments were transferred from the backbone to the methyl groups, using both scalar couplings and NOE connectivities. Subsequently, site-directed mutagenesis was employed to enable unambiguous assignment of methyl group resonances.

These assignment strategies, developed across several antibody fragments ranging from 12 to 50 kDa and by several research groups, demonstrate the feasibility of achieving near-complete methyl resonance assignments for mAb fragments (Table 4). Such assignments transform ^1^H-^13^C methyl fingerprints into powerful analytical tools capable of reporting on the HOS of antibody fragments at the residue level.

While the assignment of Fc methyl resonances for an IgG1 can be broadly applied to any IgG1 Fc fragment due to sequence similarity, the assignment of Fab-fragment methyl groups must be performed for each antibody. As illustrated in this section, obtaining a complete assignment for a Fab fragment requires substantial effort: multiple specifically labeled samples must be produced, several 3D NMR experiments acquired, and the resulting data thoroughly and often laboriously analyzed. To enable the routine use of methyl-group assignments in the development of new therapeutic antibodies, this assignment process must therefore be significantly accelerated.

### 5.3. Accelerating Methyl Assignment of IgG1 Fabs

**Figure 16:**
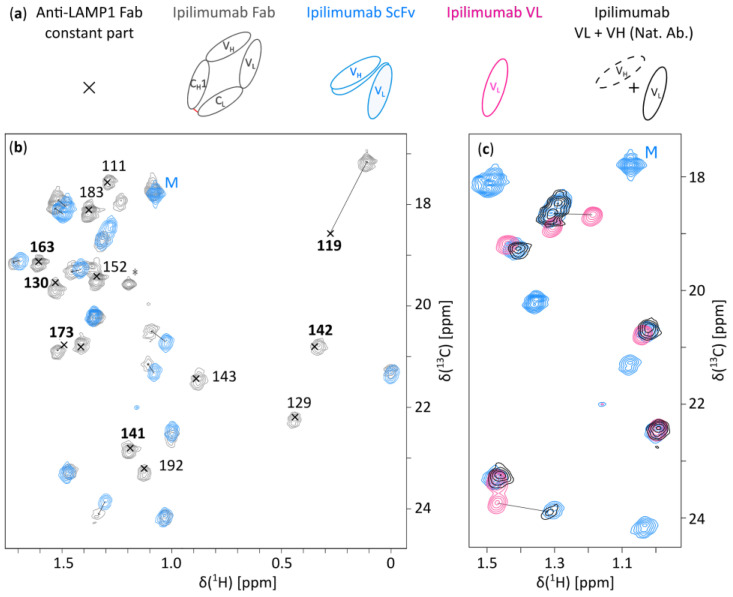
Assignment strategy shown for the alanines of ipilimumab’s Fab. All spectra displayed are from constructs labeled on Ala-[^13^CH_3_]^β^ and Met-[^13^CH_3_]^ε^. (**a**) Color-coded schemes of the different constructs for which spectra are displayed. (**b**) Superimposition of ^1^H-^13^C SOFAST methyl TROSY spectra of the Fab of ipilimumab (gray), the ScFv of ipilimumab (blue), and assigned signals from the constant part of the Fab of anti-LAMP1 (crosses). In bold are resonances belonging to the heavy chain and in regular are the ones belonging to the light chain. The asterisk indicates an impurity. (**c**) Superimposition of ^1^H-^13^C SOFAST methyl TROSY spectra of the ScFv of ipilimumab (blue), the V_L_ of ipilimumab (pink), and the V_L_ of ipilimumab to which the V_H_ fragment at natural abundance (black) has been added. Arrows indicate signal shifts between the different constructs. Signals labelled with an M correspond to a methionine. Adapted from [66].

Most therapeutic mAbs are IgG1s and therefore share not only the same Fc sequence but, in many cases, identical constant domains within their Fab fragments. This sequence identity can be exploited to accelerate Fab methyl group assignments by transferring the assignments of the constant regions from one Fab to another, leaving only the variable domains to be newly assigned for each antibody. Because these domains are much smaller than a full Fab, their resonance assignment is considerably easier: it requires less elaborate labeling schemes, reduced NMR acquisition time, and simpler data analysis. A proof of concept was demonstrated by transferring the methyl group assignments from the constant part of anti-LAMP1’s Fab to that of ipilimumab’s Fab, thereby significantly speeding up the assignment process [66]. This study showed that assignments of the Fab constant regions can be directly transferred between Fabs sharing the same constant sequence using only 2D ^1^H-^13^C methyl spectra. It also illustrated the feasibility of another “divide-and-conquer” strategy in which the scFv, or even the isolated V_H_ and V_L_ domains, can be produced and assigned separately (Figure 16).

The spectra of the V_L_, ScFv, and Fab fragments showed good overall overlap. The most significant chemical shift differences were observed between the V_L_ and ScFv spectra, for methyl groups located at the V_L_-V_H_ interface. The influence of this interface was further confirmed by adding unlabeled V_H_ to labeled V_L_ and observing the resulting chemical shift perturbations (Figure 16c). In contrast, no major shifts were detected when comparing the ScFv and the Fab spectra, indicating that the constant domains of the Fab do not induce structural changes in the variable regions (Figure 16b).

Another innovative approach has recently been proposed to significantly accelerate resonance assignments of Fab fragments [82]. By producing fragments with differential isotopic labeling—where one chain is isotopically labeled while the other remains at natural isotopic abundance—the assignment workload was drastically simplified, reducing the number of signals by a factor of two compared with the fully labeled Fab. Applying this strategy with the light chain labeled and the heavy chain unlabeled, and vice versa, with various labeling schemes, enabled assignment of backbone and methyl side-chains resonances of both Fab chains. This approach successfully reduced the total time required from initial transformation to completion of backbone assignments to approximately five weeks per chain.

The two methods developed to accelerate Fab methyl resonance assignment each have distinct advantages and limitations [66,82]. The strategy based on smaller constructs does not require deuteration and allows easier spectral analysis, but it necessitates multiple assignment transfers from VL, VH, or scFv spectra to the Fab spectrum. In contrast, the approach based on differential labeling of the two Fab chains requires the production of several deuterated samples and long NMR acquisition times due to the size of the protein. However, this method avoids chemical shift differences between the assigned constructs and the fully unlabeled Fab, unlike the smaller-construct approach. Therefore, the choice between these strategies should be guided by the available expertise and resources.

Using these strategies, Fab fragments of newly developed mAbs could be assigned within a reasonable timeframe. To leverage these assignments, obtained using isotopically enriched samples, for the analysis of NMR fingerprints of non-labeled therapeutic antibodies, a transfer of resonance assignments is required. This transfer enables residue-level HOS characterization using simple ^1^H-^13^C methyl fingerprints.

### 5.4. Transferring Methyl Assignments to Glycosylated Fc Domains

**Figure 17:**
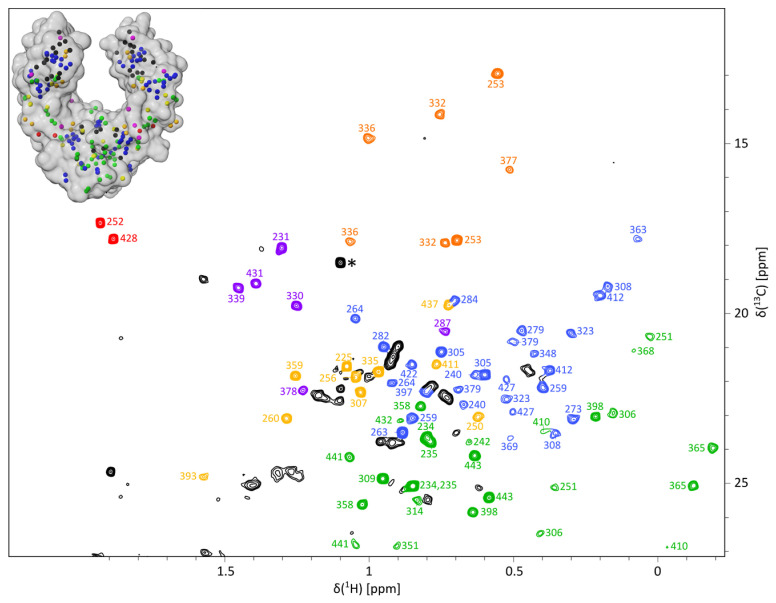
Assigned ^1^H-^13^C ALSOFAST methyl spectrum acquired on an 800 MHz spectrometer of the glycosylated Fc fragment from anti-LAMP1 mAb produced in CHO cells at natural abundance. Alanines are depicted in purple, isoleucines in orange, methionines in red, leucines in green, threonines in yellow, and valines in blue. The signal annotated with an asterisk corresponds to the methyl group of the fucose from the glycans. Top left, crystal structure of the Fc fragment (PBD 3DNK), methyl groups are represented by spheres and are colored by amino acid type if assigned. Adapted from [88].

For the Fc fragment, one assignment strategy involved using a cell-free system to produce the labeled constructs used to assign methyl group resonances [87]. This fragment differs from the Fc fragments obtained by enzymatic digestion of therapeutic IgG1s, primarily due to the presence of glycosylation. Even though the amino acid sequence is identical across all IgG1 Fc fragments, their structures, and therefore their 2D ^1^H-^13^C methyl fingerprints, can vary depending on their glycosylation state. The first near-complete assignment of methyl side-chain resonances for an IgG1 Fc fragment was achieved on a non-glycosylated Fc produced in vitro [88]. The assignment was then transferred to an Fc fragment obtained by enzymatic digestion of a glycosylated IgG1 produced in CHO cells, resulting in 83% of the methyl-containing residues being assigned (Figure 17). Although the overall 2D methyl fingerprints appear very similar, a detailed comparison of the two spectra revealed that most chemical-shift perturbations arise from the C_H_2 region, where the N-glycan is located.

This resulting fingerprint, obtained directly from an antibody expressed in CHO cells at natural abundance and under conditions identical to those used for therapeutic mAb production, shows well-resolved, minimally overlapping signals that capture structural information across the full Fc fragment (Figure 17). Thus, simple ^1^H-^13^C NMR spectra, which can be recorded within a few hours, offer a powerful approach for probing the HOS of IgG1Fc fragments at the residue level [76,88].

### 5.5. First Methyl Group Resonance Assignments of a Full IgG1

Although NMR-based HOS characterization of antibodies can be performed on Fab and Fc fragments separately, an approach that generally improves spectral quality, analyzing the HOS of a full-length mAb remains an important goal. Such fingerprints provide a more complete picture of the mAb structure and enable the study of regions such as the hinge connecting Fab and Fc domains, which cannot be characterized using individual domains. However, directly transferring assignments from isolated Fab and Fc fragments to a full-length mAb produced in CHO cells at natural isotopic abundance can be challenging due to signal overlap. Additionally, differences in buffer can induce small chemical-shift variations, further complicating the transfer of assignments.

This assignment transfer was successfully performed by Gagné and co-workers using a commercially available sample of bevacizumab at natural isotopic abundance [82]. A temperature of 50 °C was chosen for the assignment transfer to improve spectral quality of the 150 kDa full-length mAb. First, signal assignments from isotopically labeled Fab and Fc fragments obtained initially at 40 °C were transferred to spectra recorded at 50 °C by sequential spectral overlay of 2D spectra recorded at intermediate temperatures. The resulting assignments at 50 °C of methyl resonances from the light and heavy chains of the Fab were then transferred to the full-length commercial mAb by simple 2D spectral overlay. For the Fc fragment, assignments obtained for a non-glycosylated Fc were transferred to the full-length commercial mAb after enzymatic removal of glycans. The assigned spectrum of full-length bevacizumab, using methyl resonances from the Fab fragment for the glycosylated antibody and from the Fc fragment for the non-glycosylated antibody, exhibited significant overlap in the central region, which can complicate detailed analysis.

**Figure 18:**
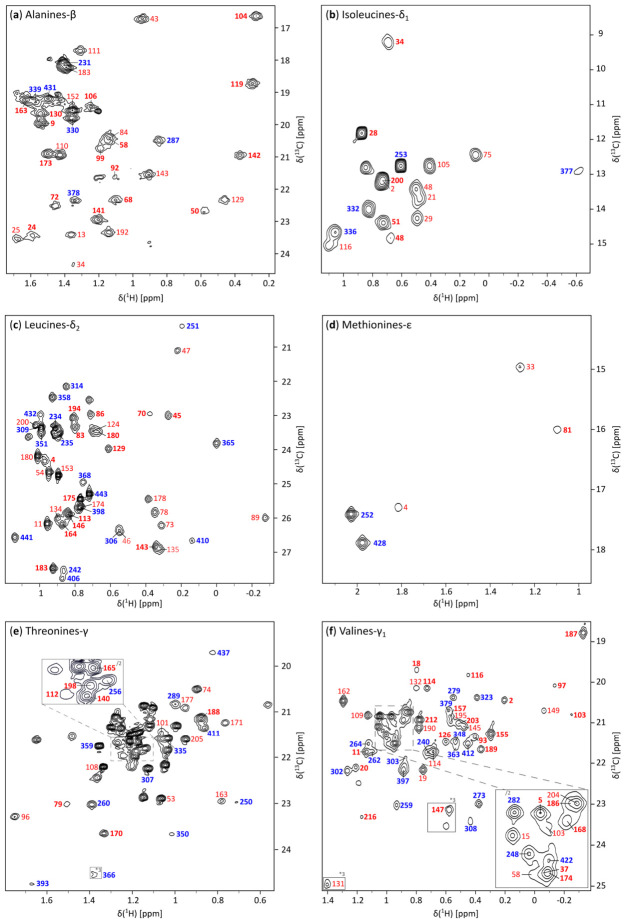
Assigned 2D ^1^H-^13^C SOFAST HMQC spectra of the anti-LAMP1 mAb produced in CHO cells and specifically labeled on (**a**) alanines-β, (**b**) isoleucines-δ_1_, (**c**) leucines-δ_2_, (**d**) methionines-ε, (**e**) threonines-γ, and (**f**) valines-γ_1_. Each assigned signal is annotated with the corresponding residue number: in blue for the Fc fragment, red for the Fab fragment, bold for the heavy chain, and normal for the light chain. In panels (**e**) and (**f**), contour levels were adjusted for some peaks, which are indicated with gray rectangles. Adapted from [75].

An alternative approach to avoid such crowded spectral regions is the isotopic labeling of full-length mAbs. The methyl group assignment of a full-length glycosylated anti-LAMP1 mAb produced in CHO cells with specific methyl group labeling was recently reported [75]. In this study, six selectively labeled samples were generated, each enriched with ^13^C at one of the following methyl groups: alanine-β, isoleucine-δ_1_, leucine-δ_2_, methionine-ε, threonine-γ, and valine-γ_1_. High-quality 2D ^1^H-^13^C SOFAST-HMQC spectra were acquired, revealing 210 well-resolved peaks out of the 220 expected signals. By overlaying these spectra with the assigned spectra of the Fab produced in a cell-free system [66] and of the Fc obtained from digestion of a CHO-expressed IgG1 [88], 184 methyl signals were successfully transferred to the spectrum of the full mAb (Figure 18).

These first assignments of 2D methyl spectra of CHO-produced mAbs represent a major milestone in achieving atomic-resolution HOS characterization of therapeutic antibodies. They now provide a reference fingerprint to directly monitor the structure of anti-LAMP1 and bevacizumab batches produced at natural abundance.

### 5.6. Towards an Atomic-Resolution Characterization of Antibody HOS at Natural Abundance

Assigned NMR methyl fingerprints of therapeutic mAbs and fragments enable localization on the 3D structure of the amino acids responsible for signal shifts observed when comparing spectra. This readout can be used to detect antigen-binding sites, characterize interactions with molecular partners, monitor structural rearrangements, and observe stress-induced modifications. During therapeutic mAb development, multiple forced degradation studies are routinely performed, and methyl NMR fingerprinting has proven to be a powerful approach to control HOS under these stresses. Among stress liabilities, methionine oxidation has been intensively investigated by NMR [48,53,106,107], and two recent studies have leveraged methyl-group resonance assignments to improve residue-level interpretation.

Previously, multivariate analysis of ^1^H-^13^C methyl fingerprints showed that, beyond methionines, additional methyl signals undergo significant shifts during forced oxidation [53]. In a collaborative study involving Bruker and the FDA, the published methyl resonance assignments for isoleucine, leucine, and valine residues of the adalimumab Fab [81] were used to map residues corresponding to signals highlighted by multivariate analysis. However, due to the limited spectral resolution of intact mAb spectra acquired at 600 MHz, only 9 out of 117 methyl-bearing residues yielded well-resolved peaks. Consequently, among the spectral features identified as discriminative between unstressed and stressed samples, one signal could be unambiguously assigned, four were ambiguously assigned to two potential residues, and the remaining signals could not be assigned [2]. Nevertheless, this first methyl fingerprint analysis of a mAb using previously reported assignments demonstrates the high added value and strong potential of methyl group assignment to obtain precise structural information from 2D fingerprints to support quality control of mAbs at natural abundance.

**Figure 19:**
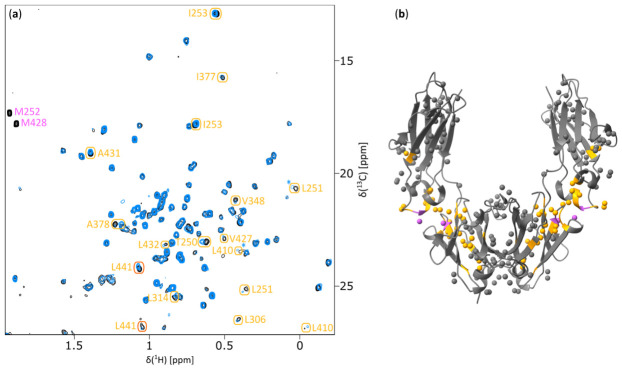
(**a**) Superimposition of ^1^H-^13^C ALSOFAST methyl spectra of the Fc fragment from anti-LAMP1 mAb before (in black) and after (in blue) methionine oxidation, recorded on an 800 MHz spectrometer at 40 °C. Orange circles indicate assigned methyl resonances for which CSP values are above 0.01 ppm. Upon oxidation, methionine signals shift outside of the spectrum window. Asterisks indicate signals for which the contour level was multiplied by a factor of 2. (**b**) Impact of methionine oxidation shown on the structure of a glycosylated Fc fragment (PBD: 3JII). Methyl groups are represented by spheres, assigned methylated amino acids with CSP ≥ 0.01 ppm are in orange and methionines are in pink. CSP values were calculated using the following formula: $CSP=\sqrt{∆\delta_{H}^{2}+{\left(\frac{∆\delta_{C}}{4}\right)}^{2}}$. Adapted from [88].

A second study focusing on the Fc fragment of an anti-LAMP1 antibody used methyl resonance assignments to identify residues responsible for spectral differences between non-oxidized and oxidized samples [88]. The non-oxidized spectrum, acquired at 800 MHz, displayed high resolution, enabling assignment of 83% of methyl signals to their corresponding residues (Figure 17). Chemical shift perturbations between native and oxidized Fc spectra were then mapped onto the Fc structure, revealing that methionine oxidation induced local rather than global effects, largely confined to residues surrounding the oxidized methionines (Figure 19). The improved resolution relative to the adalimumab study reflects both the higher magnetic field and the focus on the Fc fragment rather than the full-length mAb.

Glycosylation is another critical feature of therapeutic antibodies that must be tightly controlled during mAb development and has been extensively investigated using NMR fingerprinting. A recent study employed methyl resonance assignment to provide a site-specific interpretation of the differences observed among the spectra of the G0, G2, G0F, and G2F forms of the Fc fragment of rituximab at natural abundance [76]. Comparison of the four spectra “revealed subtle yet functionally significant Fc structural differences” [76] (p. 8379), which could be associated with core fucosylation and terminal galactosylation.

Together, these studies demonstrate the power of NMR to characterize the structure of antibodies at natural abundance, at an atomic resolution. While technical hurdles remain, most notably spectral overlap for intact mAbs at moderate fields, ^1^H-^13^C methyl fingerprinting is a sensitive and practical quality-control tool to monitor the structural integrity of therapeutic mAbs throughout development, particularly when leveraged with resonance assignments, high magnetic fields, and fragment analyses.

### 5.7. Towards Structure Determination and Dynamic Studies of Antibody Fragments Using NMR

Beyond atomic-resolution characterization of antibody HOS, amide backbone and methyl side-chain assignments paves the way for precise interpretation of a wide range of NMR experiments that can be recorded on isotopically labeled antibody fragments. Among these, paramagnetic relaxation enhancement (PRE) NMR provides long-range distance information and sensitivity to low-populated transient states, making it particularly powerful for studying antibody–antigen interactions. This methodology has been used on two labeled scFv constructs [98,108]. PRE measurements revealed the presence of transient interactions between the scFv of an anti-MSP2 mAb and its disordered antigen [98]. In addition, it enabled determination of epitope orientation within the scFv construct of an anti-prion antibody, offering structural insight into paratope-epitope organization [108].

Although the majority of antibody fragment structures have been determined by X-ray crystallography, solution NMR also offers the opportunity to determine three-dimensional solution structures. For more details on the methodological aspects of NMR structure calculation, we refer the reader to dedicated reviews [109,110], and will focus here on selected examples of antibody fragment structures determined by NMR.

For instance, the solution structure of the binding site of the anti-HIV-1 0.5β Fv in complex with the V3 peptide P1053 was determined using 1138 distance restraints and 80 dihedral restraints, revealing the detailed interactions between the antibody CDRs and the viral epitope [111]. Complete structures of isolated VH domains have also been solved, such as the llama VH domain BrucD4-4 [112], and the camelised human VH domain VH-P8 [113]. Moreover, the solution structure of an isolated VL domain from anti-digoxin antibody 26-10 demonstrated that this domain retains its native fold even in the absence of its VH partner [114].

A unique advantage of solution NMR over X-ray crystallography for studying antibody–antigen interactions is the ability to use transferred NOE experiments. This approach is particularly valuable when studying peptide antigens or small-molecule ligands that bind to antibodies with moderate affinity, leading to rapid exchange between free and bound states. For instance, Anglister and colleagues applied 2D transferred NOE difference spectroscopy to investigate the interactions between three anti-cholera toxin peptide antibodies (TE32, TE33 and TE34) and their peptide antigen CTP3. This methodology enabled identification of specific antibody-peptide interactions and revealed that TE32 and TE33 show similar interaction patterns with CTP3, while TE34 exhibits a different binding mode with the same peptide [115,116].

Complementary to structural studies, NMR relaxation measurements provide valuable insights into antibody dynamics. Backbone ^15^N relaxation parameters (T_1_, T_2_, T_1_ρ, heteronuclear NOE, and CPMG) can probe domain flexibility and identify regions undergoing conformational exchange. For instance, relaxation studies of the llama VH domain BrucD4-4 revealed increased mobility in the CDR loops compared to the well-folded β-barrel core, with heteronuclear NOE values around 0.8 for β-strand residues but significantly lower (0.4–0.7) for the loop regions connecting the β-strands, indicating that the CDR loops were more flexible [112]. More recently, comprehensive NMR studies of the anti-IL-1β Fab and anti-IL-6 scFv fragments revealed that the hearts of the antigen-binding sites exist in multiple conformations interconverting on millisecond-to-second timescales, as evidenced by the absence of backbone amide signals for many CDR3 residues in the free antibodies [117]. This conformational exchange was significantly reduced upon antigen binding, with many previously unobservable CDR signals appearing in the spectra of the antibody–antigen complexes, suggesting a stabilization of the binding site through target protein interactions.

While complete structure determination of full-length mAbs remains challenging due to their size and complexity, NMR-derived structural and dynamic information on antibody fragments provides valuable complementary data to other structural biology tools such as cryo-EM and X-ray crystallography, particularly for characterizing flexible regions, conformational exchange processes and solution-state conformations relevant to antibody function.

## 6. Conclusions

Since the late twentieth century, monoclonal antibodies have been extensively studied due to their central role in immune responses. In many therapeutic areas, they have emerged as promising candidates to treat previously incurable diseases or to replace existing therapies with safer and more effective biomolecules. mAbs are large proteins composed of four polypeptide chains that assemble into a well-defined three-dimensional architecture. The higher-order structure is essential for antigen recognition as well as for interactions with immune cell receptors. Assessing HOS throughout mAb development is therefore critical to ensure that biological activity and safety remain uncompromised during storage, process changes, formulation optimization, or stress testing.

In recent years, solution-state NMR has emerged as a powerful tool for characterizing mAb HOS during therapeutic development. Although historically limited to low-molecular-weight proteins, NMR offers atomic-resolution structural insights with minimal sample preparation. This review highlights recent advances in NMR methodologies adapted to therapeutic antibody analysis. These approaches include the development of optimized NMR experiments and the production of isotopically labeled mAbs, which have collectively enabled the first assignments of the backbone and methyl NMR signals of Fab and Fc fragments, as well as full-length mAbs. These results establish methyl-group NMR fingerprinting as the most effective NMR strategy for detecting HOS differences in mAbs at atomic resolution. This pioneering work paves the way for the integration of NMR into pharmaceutical quality control, offering a sensitive technique capable of detecting HOS changes at atomic resolution under conditions closely matching formulation buffers and at natural isotopic abundance.

## Figures and Tables

**Table 1:** Isotopic labeling strategies used for NMR studies of mAbs and their fragments.

Labeling Scheme	Application
^13^C, ^15^N	Backbone and methyl assignment (small fragments ^1^)
^2^H, ^13^C, ^15^N	Backbone assignment (large fragments ^1^)
Uniform ^15^N	Amide fingerprinting
Uniform ^13^C	Methyl fingerprinting
Fractional ^13^C	Stereospecific assignment (Leu, Val)
Specific ^13^CH_3_ +/− ^2^H	Methyl assignment and fingerprinting
Amino acid-specific	Simplified spectra, reduced overlap
Stereospecific ^13^CH_3_	Stereospecific methyl assignment

^1^ Fragment size classification is based on the rotational correlation time (τ_c_), which depends on both molecular size and temperature. Hence, ScFv, VL, VH and Fc fragments can be considered as small fragments and Fab as large ones.

**Table 2:** Summary of the advantages, disadvantages and relative costs of the four expression systems.

Expression System	Advantages	Disadvantages	Production Timescale ^1^	Relative Isotope Cost
CHO cells	Native PTMs Glycosylated mAbs High antibody yields	Perdeuteration not possible Limited labeling flexibility	Few weeks	€€€
*E. coli*	High deuteration levels Broad labeling strategies	No glycosylation Extensive refolding process	Few days	€ (^15^N, ^13^C) €€ (^15^N, ^13^C, ^2^H)
Pichia pastoris yeast	No refolding required N-glycosylation (non-human) Tolerates high levels of D_2_O	Low yields with deuteration Unfolding needed for amide proton back-exchange	One week	€ (^15^N, ^13^C) €€ (^15^N, ^13^C, ^2^H)
Cell-free (*E. coli*)	Unlimited labeling flexibility No refolding required Broad labeling strategies	No glycosylation	One day	€ (^15^N, ^13^C) €€ (^15^N, ^13^C, ^2^H)

^1^ The indicated times correspond to the production of non-deuterated proteins.

**Table 4:** List of the BMRB deposition numbers corresponding to methyl group assignments of mAb fragments.

BMRB Number	Assigned Fragment	Assigned Methyl Groups ^1^	Publication Year
15204 [99]	Reduced C_H_3 domain IgG1	A, I^δ1, γ2^, L *, M, T, V *	2007
51094 [49]	scFv NISTmAb	A, I^δ1, γ2^, L *, M, T, V *	2022
52228 [80]	Sc-Fab trastuzumab	I^δ1^, L^δ1, δ2^, V^γ1, γ2^	2024
52274 [81]	Fab adalimumab	I^δ1^, L *, V *	2024
53197 [82]	Fc non-glycosylated (IgG1-EEM)	A, I^δ1^, L *, T, V *	2026
53199 [82]	Fc non-glycosylated (IgG1-DEL)	A, I^δ1^, L *, T, V *	2026
53403 [76]	Fc IgG1 G0F	A, I^δ1, γ2^, L^δ1, δ2^, T, V^γ1, γ2^	2026
53420 [66]	V_L_ ipilimumab	A, I^δ1, γ2^, L^δ1, δ2^, M, T, V^γ1, γ2^	2026
53421 [66]	ScFv ipilimumab	A, I^δ1, γ2^, L^δ1, δ2^, M, T, V^γ1, γ2^	2026
53422 [66]	Fab ipilimumab	A, I^δ1, γ2^, L^δ1, δ2^, M, T, V^γ1, γ2^	2026
53423 [66]	Fab Anti-LAMP1	A, I^δ1^, L^δ2^, M, T, V^γ1^	2026
53440 [82]	Fab (IgG1, bevacizumab)	A, I^δ1, γ2^, L *, T, V *	2026
53464 [88]	Fc IgG1 non-glycosylated	A, I^δ1, γ2^, L *, M, T, V *	2026
53465 [88]	Fc IgG1 glycosylated	A, I^δ1, γ2^, L *, M, T, V *	2026
53491 [82]	Fab (IgG1, rituximab)	I^δ1^, L *, V *	2026
53625 [82]	Fc G0, G1, G2 (IgG1-EEM)	A, I^δ1^, L *, T, V *	2026

^1^ Asterisks indicate that assignment of leucine or valine residues is not stereospecific. For the Fab of ipilimumab, the assignment of I^γ2^, L^δ1^, V^γ2^ were obtained only for the variable region.

## Data Availability

No new data were created or analyzed in this study.

## Abbreviations

The following abbreviations are used in this manuscript:

ADCC	Antibody-Dependent Cellular Cytotoxicity
ADCP	Antibody-Dependent Cellular Phagocytosis
CD	Circular Dichroism
CDC	Complement-Dependent Cytotoxicity
CHO	Chinese Hamster Ovary
COSY	COrrelation SpectroscopY
DLS	Dynamic Light Scattering
DSC	Differential Scanning Calorimetry
EM	Electron Microscopy
ER	Endoplasmic Reticulum
Fab	Fragment antigen-binding
Fc	Fragment crystallizable
FDA	Food and Drug Administration
FTIR	Fourier-Transform Infrared
HDX	Hydrogen-Deuterium Exchange
HMQC	Heteronuclear Multiple Quantum Coherence
HOS	Higher Order Structure
HSQC	Heteronuclear Single Quantum Coherence
LAMP1	Lysosomal-Associated Membrane Protein 1
LLPS	Liquid–Liquid Phase Separation
mAb	monoclonal Antibody
MS	Mass Spectrometry
NIST	National Institute of Standards and Technology
NMR	Nuclear Magnetic Resonance
NOESY	Nuclear Overhauser Effect SpectroscopY
NUS	Non-Uniform Sampling
PCA	Principal Component Analysis
PROFILE	PROtein FIngerprint by Line shape Enhancement
QA	Quality Attribute
ScFv	Single-chain Fragment variable
SOFAST	Selective Optimized-Flip-Angle Short Transient
TOCSY	TOtal Correlation SpectroscopY
TROSY	Transverse Relaxation-Optimized SpectroscopY
